# Greenhouse gas emissions from municipal wastewater treatment facilities in China from 2006 to 2019

**DOI:** 10.1038/s41597-022-01439-7

**Published:** 2022-06-16

**Authors:** Dan Wang, Weili Ye, Guangxue Wu, Ruoqi Li, Yuru Guan, Wei Zhang, Junxia Wang, Yuli Shan, Klaus Hubacek

**Affiliations:** 1 Integrated Research on Energy, Environment and Society (IREES), Energy Sustainability Research Institute Groningen (ESRIG), University of Groningen, Groningen, 9747 AG The Netherlands; 2 The Center for Beijing-Tianjin-Hebei Regional Environment and Ecology, Chinese Academy of Environmental Planning, Beijing, 100012 China; 3 Civil Engineering, School of Engineering, College of Science and Engineering, National University of Ireland, Galway, Galway, H91 TK33 Ireland; 4 State Key Laboratory of Pollution Control and Resource Reuse, School of the Environment, Nanjing University, Nanjing, 210023 China; 5 State Environmental Protection Key Laboratory of Environmental Planning and Policy Simulation, Chinese Academy of Environmental Planning, Beijing, 100012 China; 6 State Environmental Protection Key Laboratory of Quality Control in Environmental Monitoring, China National Environmental Monitoring Centre, Beijing, 100012 China

**Keywords:** Environmental impact, Climate-change mitigation

## Abstract

Wastewater treatment plants (WWTPs) alleviate water pollution but also induce resource consumption and environmental impacts especially greenhouse gas (GHG) emissions. Mitigating GHG emissions of WWTPs can contribute to achieving carbon neutrality in China. But there is still a lack of a high-resolution and time-series GHG emission inventories of WWTPs in China. In this study, we construct a firm-level emission inventory of WWTPs for CH_4_, N_2_O and CO_2_ emissions from different wastewater treatment processes, energy consumption and effluent discharge for the time-period from 2006 to 2019. We aim to develop a transparent, verifiable and comparable WWTP GHG emission inventory to support GHG mitigation of WWTPs in China.

## Abstract (summary)

**Table:** 

Measurement(s)	methane, nitrous oxide and carbon dioxide emissions from municipal wastewater treatment facilities
Technology Type(s)	computational modeling technique
Factor Type(s)	influent and effluent chemical oxygen demand (COD) mass • influent and effluent total nitrogen (TN) mass • removed COD • electricity consumption • emisison factors • wastewater treatment technology
Sample Characteristic - Organism	None
Sample Characteristic - Environment	wastewater treatment facilities • physical treatment process • chemical treatment process • physicochemical treatment process • conventional activated sludge • enhanced activated sludge process • biofilm treatment process • anaerobic biological treatment process • stabilization pond, constructed wetland and land treatment • membrane bioreactor
Sample Characteristic - Location	China

## Background & Summary

Municipal wastewater treatment facilities are the main technical solution to mitigating water pollution. But wastewater purification in WWTPs and other treatment facilities always comes at the cost of energy consumption, use of chemicals and environmental impacts^1,2^, among which, GHG emissions are of most concern^3,4^. Even though GHG emissions from wastewater make only a small contribution to global anthropogenic GHG emissions, it is still important to map GHG emissions from wastewater treatment systems, and to set reasonable targets for mitigation of GHG emissions^5,6^. To achieve these purposes, a comprehensive GHG inventory is a prerequisite. There have been numerous studies establishing GHG accounts of WWTPs^7–13^, but there still exist challenges and problems.

Current GHG accounts often do not consider differences of treatment processes/technologies. The accounting of GHG emissions of WWTPs at the regional level mainly uses IPCC emission factors, where the centralized biological treatment processes are only categorized into aerobic and anaerobic processes but neglect the differentiation of sub-categories of aerobic or anaerobic technologies^7,10–14^, leading to large uncertainties of GHG emission factors. To accurately account GHG emissions in WWTPs, detailed processes/technologies should be considered and analysed.

Frequently, only CH_4_ and/or N_2_O are accounted for, excluding CO_2_ emissions of biological treatment processes as ‘these are generally derived from modern (biogenic) organic matter in human excreta or food waste and should not be included in national total emissions (IPCC 2019, Volume 5, Chapter 6, Page 7)’^15^. But intensive research has shown that a significant amount of fossil CO_2_ are directly emitted from WWTPs, and assuming that all direct CO_2_ emissions are biogenic may underestimate GHG emissions^16–20^.

Dissolved GHG in the treated effluent themselves have the potential to be released. In addition, many waterways are in eutrophic or nutrient-rich conditions, which can further induce discharged wastewater to increase GHG emissions^15^. However, GHG emissions from receiving waters are rarely accounted for, due to a lack of data of the water quality of the recipient body of water and downstream discharge pathways. Even though some studies considered off-site emissions from the treated effluent, only one discharge pathway of entering rivers, lakes or oceans was assumed^7–9^. To account emissions from different discharge pathways (such as direct discharge into rivers, lakes, reservoirs, seas, soil, and sewage irrigated farmland) is essential for identifying key emission sources, GHG composition and their contribution to the whole wastewater treatment system.

Existing reginal- or national-level studies on GHG emissions accounting of wastewater treatment systems are not comparable. This is mainly due to different emission factors and data sources in different studies. For example, Zhao, *et al*.^10^ used firm-level activity data and IPCC 2006 emission factors to calculate CH_4_ emissions, while emission factors of Yan, *et al*.^11^ were obtained from the average of four references excluding IPCC emission factors, and provincial-level activity data from China Environment Yearbook and China Statistical Yearbook. Differences in applied methodology and data sources contribute to a factor 38 difference in calculated CH_4_ emissions for the same year.

To solve the above gaps, we constructed a high-resolution (firm-level) and time series (from 2006 to 2019) GHG emission inventory of WWTPs in China. Emission sources include on-site emissions from biological treatment processes and off-site emissions from energy consumption and discharge pathways of the WWTP. We distinguished between 10 potential pathways: direct and indirect (after sewers) discharge into seas; direct and indirect discharge into rivers, lakes, reservoirs etc.; municipal WWPTs; direct discharge onto sewage irrigated farmlands; discharge onto land; other facilities (decentralized wastewater treatment facilities); centralized industrial WWTPs and other discharge pathways. To account for the different emission potentials of different treatment technologies, we calculated emissions based on 48 separate biological, physical, chemical and physicochemical technologies and their combinations. GHG emission factors of different biological treatment technologies in line with China’s conditions were obtained from the literature. Three GHG were estimated in this research, i.e., CO_2_, N_2_O and CH_4_. We did not distinguish between fossil CO_2_ and biogenic CO_2_ emissions from biological treatment but regarded CO_2_ emission as the sum of fossil CO_2_ and biogenic CO_2_ emissions.

## Methods

We include GHG emissions from domestic wastewater treated by municipal WWTPs and other facilities in this paper. The other facilities mainly collect and treat wastewater discharged from residential areas, tourist facilities, resorts, nursing homes, airports, railway stations, and other public places. Domestic wastewater collected by both municipal WWTPs, and other facilities maybe mixed with industrial wastewater under certain conditions. In this case, IPCC 2019 suggests that the mixed domestic and industrial wastewater can be considered as domestic wastewater^15^.

GHG emissions of a WWTP result from on-site and off-site emissions. On-site emissions are usually defined as emissions induced by wastewater and sludge treatment processes of WWTPs^21,22^. In our study, the system boundary excludes GHG emissions from sludge treatment and disposal processes in a WWTP due to lack of data, even though it is reported that sewage sludge treatment and disposal processes account for about 40% GHG emissions in wastewater systems^23^. On the other hand, generated CH_4_ emissions from a WWTP are rarely recovered or flared in China, we regard recovered or flared CH_4_ emissions as being zero. Therefore, on-site emissions only refer to emissions from wastewater treatment procedures in this research. For various wastewater treatment technologies, biological treatment technologies generate on-site GHG emissions during wastewater treatment processes, but physical, chemical, and physicochemical treatment technologies do not. Off-site emissions refer to emissions from effluent, electricity consumption, production and transportation of chemicals. But we exclude off-site emissions generated by chemicals’ production and transportation due to lack of data for each WWTP, and they being negligible compared with electricity consumption^13^. CO_2_ emissions from electricity consumption are fossil CO_2_, because they come from coal-fired power generation, but CO_2_ emissions generated by on-site wastewater treatment and off-site effluent are mixed with fossil CO_2_ and biogenic CO_2_, as influent and effluent COD may contain both fossil and biogenic carbon.

**Fig. 1:**
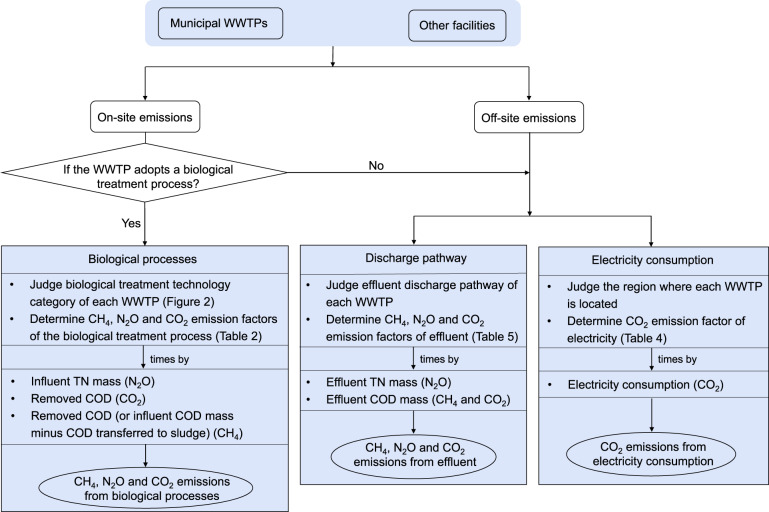
The flowchart of the construction of firm-level GHG emissions inventory of wastewater treatment facilities from 2006 to 2019 in China. Only biological treatment processes emit on-site GHG, but physical, chemical, and physicochemical treatment technologies do not generate on-site GHG emissions.

Figure 1 shows a flowchart of the construction of the firm-level GHG emission inventory of wastewater treatment facilities from 2006 to 2019 in China. The first step to quantify GHG emissions of a WWTP is to judge the applied treatment technology. If the WWTP adopts a biological process, on-site emissions from the biological treatment process are calculated. Otherwise, off-site emissions from electricity consumption and the discharge pathway for each WWTP are quantified. Calculation of GHG emissions from each emission source was based on the multiplication of emission factors and activity data. The activity data for each WWTP was collected from China Environmental Statistics Database (CESD)^24–37^.

### Classification of wastewater treatment technologies and its priority

**Fig. 2:**
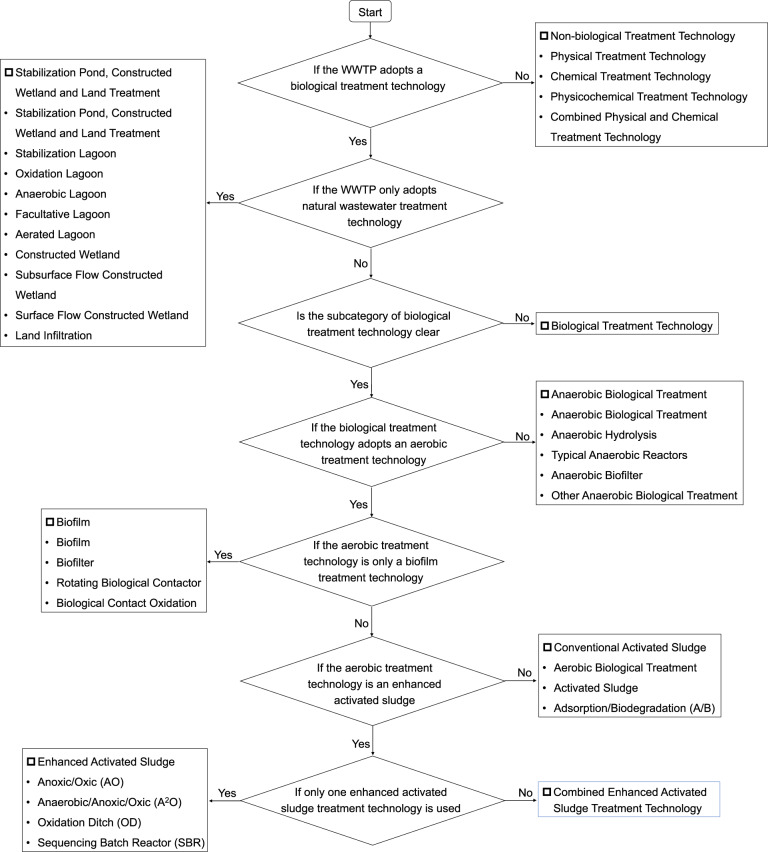
A decision tree for determining the category of treatment technology of a WWTP.

To examine GHG emissions of different wastewater treatment processes, we need to decide the category of technology applied in each WWTP. In most cases, a WWTP has a primary, secondary or tertiary treatment process, and for each process, especially in secondary treatment, more than one technology may be applied. It is impossible to quantify on-site GHG emissions for each technology, since we only collected data on concentration of influent and effluent pollutants for the whole WWTP, rather than for each technology or process. Therefore, to simplify the calculations of on-site GHG emissions, we first need to judge the main category of treatment technology of a WWTP, and then choose the corresponding emission factors of CH_4_, N_2_O and CO_2_ to calculate GHG emissions generated by biological treatment processes. The technology classification is presented in Table 1. A decision tree for determining the category of treatment technology of a WWTP is shown in Fig. 2.

**Table 1:** Classification of treatment processes of WWTPs in China .

No.	Treatment processes	No.	Treatment processes
**1**	**Physical Method**	5.1	Anoxic/Oxic (AO)
1.1	Physical Treatment	5.2	Anaerobic/Anoxic/Oxic (A^2^O)
1.2	Filtration and Separation	5.3	Oxidation Ditch (OD)
1.3	Membrane Separation	5.4	Sequencing Batch Reactor (SBR)
1.4	Centrifugal Separation	**6**	**Biofilm**
1.5	Settlement	6.1	Biofilm
1.6	Flotation Separation	6.2	Biofilter
1.7	Evaporation Crystallization	6.3	Rotating Biological Contactor
1.8	Other Physical Treatment	6.4	Biological Contact Oxidation
**2**	**Chemical Method**	**7**	**Anaerobic Biological Method**
2.1	Chemical Treatment	7.1	Anaerobic Biological Treatment
2.2	Neutralization	7.2	Anaerobic Hydrolysis
2.3	Chemical Precipitation	7.3	Typical Anaerobic Reactors
2.4	Oxidation Reduction	7.4	Anaerobic Biofilter
2.5	Electrolysis	7.5	Other Anaerobic Biological Treatment
2.6	Other Chemical Treatment	**8**	**Stabilization Pond, Constructed Wetland and Land Treatment**
**3**	**Physicochemical Method**	8.1	Stabilization Pond, Constructed Wetland and Land Treatment
3.1	Physicochemical Treatment	8.2	Stabilization Lagoon
3.2	Chemical Coagulation	8.3	Oxidation Lagoon
3.3	Adsorption	8.4	Anaerobic Lagoon
3.4	Ion Exchange	8.5	Facultative Lagoon
3.5	Electrodialysis	8.6	Aerated Lagoon
3.6	Other Physicochemical Treatment	8.7	Constructed Wetland
**4**	**Conventional Activated Sludge**	8.8	Subsurface Flow Constructed Wetland
4.1	Aerobic Biological Treatment	8.9	Surface Flow Constructed Wetland
4.2	Activated Sludge	8.10	Land Infiltration
4.3	Adsorption/Biodegradation (A/B)	**9**	**Membrane Bioreactor (MBR)**
**5**	**Enhanced Activated Sludge Process**	**10**	**Biological Treatment**

Note: Wastewater treatment technologies of Conventional Activated Sludge (4), Enhanced Activated Sludge Process (5), Biofilm (6), Anaerobic Biological Method (7), Stabilization Pond, Constructed Wetland and Land Treatment (8) all belong to subcategories of biological treatment processes. But for some WWTPs, their subcategories of biological treatment processes were not reported in the original dataset. In this case, their treatment technologies were named as Biological Treatment (10), and their GHG emissions are estimated by emission factors of the technology of activated sludge treatment (4.2 in Table 1), as it is recognized as the most popular wastewater treatment technology around the world.

### On-site emissions from biological treatment

#### CH_4_, N_2_O and CO_2_ emissions estimated by this study

WWTPs or other treatment facilities, which have biological treatment processes, emit CH_4_, N_2_O and CO_2_ directly, which were calculated by Eq. 1.1, 1.2 and 1.3, respectively. The CH_4_, N_2_O and CO_2_ emission factors of different biological treatment processes adopted in this study were obtained from the literature, and most were studies on GHG emission factors of existing Chinese WWTPs. On the other hand, some emission factors were adopted from the IPCC 2019 report, laboratory-based studies or other models, because of a lack of studies on emission factors of full-scale wastewater treatment processes. Detailed CH_4_, N_2_O and CO_2_ emission factors from the literature were summarised in Table S1, Table S2 and Table S3, respectively. We obtained the minimum, maximum and average values of emission factors for each biological treatment process. The average values of emission factors were defined as the default emission factors in this study, and they are shown in Table 2. We also list IPCC 2019 emission factors of biological treatment processes in Table 2 for comparison. For those WWTPs or other treatment facilities adopted by the combined enhanced activated sludge treatment technology, their emissions factors are mean of emission factors of specific enhanced activated sludge treatment technologies (i.e., AO, A^2^O, OD or SBR). CH_4_ and N_2_O are converted to the CO_2_ equivalent by Global Warming Potential (GWP) values for 100 years. The GWP of N_2_O, CH_4_ and CO_2_ are 265, 28 and 1, respectively^38^.

11 $$\begin{array}{c}CH{4}_{bio,i}=E{F}_{bio,CH4,j}\ast A{D}_{bio,CH4,i}\ast 28\end{array}$$

12 $$\begin{array}{c}N2{O}_{bio,i}=E{F}_{bio,N2O,j}\ast T{N}_{in,i}\ast 265\end{array}$$

13 $$\begin{array}{c}CO{2}_{bio,i}=E{F}_{bio,CO2,j}\ast CO{D}_{removed,i}\end{array}$$

**Table 2:** Default GHG emission factors of biological treatment technologies in this study and in IPCC 2019.

No.	Biological treatment technology	IPCC 2019		This study		
		CH_4_	N_2_O	CH_4_	N_2_O	CO_2_
		g CH_4_/kg COD	g N_2_O /kg TN influent	g CH_4_/kg COD removed (No. 1–11); g CH_4_/kg COD (No, 12–27)	g N_2_O /kg TN influent	g CO_2_/kg COD removed
1	Aerobic Biological Treatment	7.50	25.00	0.70	1.20	560.00
2	Activated sludge	7.50	25.00	0.70	1.20	560.00
3	AO	7.50	25.00	0.74	13.94	365.75
4	A^2^O	7.50	25.00	2.66	6.19	375.53
5	OD	7.50	25.00	4.27	2.18	510.65
6	SBR	7.50	25.00	1.76	43.60	531.80
7	AB	7.50	25.00	0.70	1.20	560.00
8	Biofilm	0.00	25.00	0.00	11.67	436.20
9	Biofilter	0.00	25.00	0.00	11.67	436.20
10	Rotating Biological Contactor	0.00	25.00	0.00	11.67	436.20
11	Biological Contact Oxidation	0.00	25.00	0.00	11.67	436.20
12	Anaerobic Biological Treatment	200.00	0.00	200.00	0.00	380.50
13	Anaerobic Hydrolysis	200.00	0.00	200.00	0.00	380.50
14	Typical Anaerobic Reactors	200.00	0.00	200.00	0.00	380.50
15	Anaerobic Biofilter	200.00	0.00	200.00	0.00	380.50
16	Other Anaerobic Biological Treatment	200.00	0.00	200.00	0.00	380.50
17	Stabilization Pond, Constructed Wetland and Land Treatment	68.06	11.98	68.06	11.98	502.91
18	Stabilization Lagoon	66.25	18.75	66.25	18.75	515.13
19	Oxidation Lagoon	7.50	25.00	7.50	25.00	560.00
20	Anaerobic Lagoon	200.00	0.00	200.00	0.00	380.50
21	Facultative Lagoon	50.00	25.00	50.00	25.00	560.00
22	Aerated Lagoon	7.50	25.00	7.50	25.00	560.00
23	Constructed Wetland	42.50	4.94	42.50	4.94	482.54
24	Subsurface Flow Constructed Wetland	13.75	6.39	13.75	6.39	482.54
25	Surface Flow Constructed Wetland	100.00	2.04	100.00	2.04	482.54
26	Land Infiltration	125.00	0.70	125.00	0.70	502.91
27	Biological Treatment	7.50	25.00	7.50	25.00	560.00

Note: The CH_4_, N_2_O and CO_2_ emission factors of different biological treatment processes adopted in this study were obtained from the literature. Some emission factors were from studies on GHG emission factors of Chinese WWTPs. However, because of a lack of studies on emission factors of full-scale wastewater treatment processes in China, emission factors of some specific treatment technologies were adopted from the IPCC 2019 report (CH_4_ and N_2_O emission factors of anaerobic biological treatment processes (12–16) and stabilization pond, constructed wetland and land treatment method (17–26)), laboratory-based studies (N_2_O emission factors of biofilm processes (8–11)) or other models (CO_2_ emission factors of aerobic biological treatment process (1), activated sludge process (2), biofilm processes (8–11), and CO_2_ and CH_4_ emission factors of anaerobic biological treatment processes (12–16)).

Where, $CH{4}_{bio,i}$, $N2{O}_{bio,i}$ and $CO{2}_{bio,i}$ refer to CH_4_, N_2_O, and CO_2_ emissions (g CO_2_eq/year) from biological treatment processes in the *i* th WWTP. $E{F}_{bio,CH4,j}$ (g CH_4_/kg COD removed or g CH_4_/kg COD), $E{F}_{bio,N2O,j}$ (g N_2_O/kg TN influent) and $E{F}_{bio,CO2,j}$ (g CO_2_/kg COD removed) are three GHG emission factors of the process *j* in the *i* th WWTP. $A{D}_{bio,CH4,i}$ is activity data of biological CH_4_ emissions. There are two types of $A{D}_{bio,CH4,i}$. When the unit of $E{F}_{bio,CH4,j}$ for the process *j* is g CH_4_/kg COD removed, $A{D}_{bio,CH4,i}$ is the removed COD per year (kg COD removed/year) in the *i*th WWTP. But $A{D}_{bio,CH4,i}$ refers to the difference between influent COD mass and COD transferred to sludge if the unit of $E{F}_{bio,CH4,j}$ is g CH_4_/kg COD. In the section of ‘Calculation of COD removed in the form of sludge’, we described how to estimate the COD transferred in the form of sludge for each process. $T{N}_{in,i}$ is the annually influent TN mass (kg TN influent/year) in the *i*th WWTP, and $CO{D}_{removed,i}$ is the annually removed COD (kg COD removed/year) in the *i*th WWTP.

#### CH_4_ and N_2_O emissions estimated by IPCC 2019

To make a comparison with our study, we also used the method of IPCC 2019 to calculate CH_4_ and N_2_O emissions from biological treatment processes. CH_4_ and N_2_O emission factors for each wastewater treatment process are from IPCC 2019 (Table 2).

14 $$\begin{array}{c}CH{4}_{IPCC\_bio,i}=[E{F}_{IPCC\_bio,CH4,j}\ast (CO{D}_{in,i}-{S}_{COD,i})-{R}_{CH4,i}]\ast 28\end{array}$$

15 $$\begin{array}{c}N2{O}_{IPCC\_bio,i}=E{F}_{IPCC\_bio,N2O,j}\ast T{N}_{in,i}\ast 265\end{array}$$

where, $CH{4}_{IPCC\_bio,i}$ and $N2{O}_{IPCC\_bio,i}$ refer to CH_4_ and CO_2_ emissions (g CO_2_eq/year) from biological treatment processes in the *i*th WWTP. $E{F}_{IPCC\_bio,CH4,j}$ (g CH_4_/kg COD) and $E{F}_{IPCC\_bio,N2O,j}$ (g N_2_O/kg TN influent) are IPCC 2019 CH_4_ and N_2_O emission factors of the process *j* in the *i* th WWTP. $CO{D}_{in,i}$ is the annually influent COD mass (kg COD influent/year) in the *i*th WWTP. ${S}_{COD,i}$ (kg COD removed as sludge/year) is the COD removed in the form of sludge in the *i*th WWTP. ${R}_{CH4,i}$ is amount of CH_4_ recovered or flared from the *i*th WWTP. This value was regarded as being zero because there are very few CH_4_ recovered or flared in China. $T{N}_{in,i}$ is the annually influent TN mass (kg TN influent/year) in the *i* th WWTP.

#### Calculation of COD removed in the form of sludge

16 $$\begin{array}{c}{S}_{COD,i}=CO{D}_{removed,i}\ast {Y}_{obs,j}\ast 1.42\end{array}$$

17 $$\begin{array}{c}CO{D}_{removed,i}=(CO{D}_{in}-CO{D}_{out})\ast {V}_{wastewater,i}\end{array}$$

where, ${S}_{COD,i}$ (g COD removed as sludge/year) is the COD removed in the form of sludge in the *i*th WWTP, $CO{D}_{removed,i}$ (g COD/year) is the COD removed of the *i*th WWTP. ${Y}_{obs,j}$ (g VSS/ g COD) is the observed sludge yield of process *j* in the *i*th WWTP. 1.42 (g COD/ g VSS) is the conversion factor that determine biomass concentration in terms of COD^39^. $CO{D}_{in}$ and $CO{D}_{out}$ are influent and effluent COD concentration of the *i*th WWTP. ${V}_{wastewater}$ is the volume of treated wastewater in the *i*th WWTP. The coefficient of ${Y}_{obs,j}$ (g VSS/ g COD) for each process is from *Chen et al*.^40^. Since a membrane bioreactor (MBR) is the combination of an enhanced activated sludge process and a membrane process, its ${Y}_{obs,j}$ was estimated by the average value of observed sludge yield of an enhanced activated sludge process and a biofilm process. Coefficients ${Y}_{obs,j}$ of different treatment processes are shown in Table 3.

**Table 3:** Coefficients *Y*_*obs*_ of biological treatment processes.

Process	*Y*_*obs*_ (g VSS/g COD)
Conventional Activated Sludge	0.350
Biofilm	0.250
Anaerobic Biological Treatment	—
Stabilization Pond, Constructed Wetland and Land Treatment	—
Biological treatment	0.350
AO	0.290
A^2^O	0.290
OD	0.220
SBR	0.260
AO + A^2^O	0.290
AO + OD	0.255
AO + SBR	0.275
AO + MBR	0.270
A^2^O + OD	0.255
A^2^O + SBR	0.275
A^2^O + MBR	0.270
OD + SBR	0.240
OD + MBR	0.235
SBR + MBR	0.255

Note: Coefficients *Y*_*obs*_ of Anaerobic Biological Treatment processes and Stabilization Pond, Constructed Wetland and Land Treatment processes were not considered in this study, as they are relatively lower or more difficult to obtain compared with other biological treatment processes. Coefficients *Y*_*obs*_ of combined enhance activated sludge treatment technology in this study are the average *Y*_*obs*_ of specific enhance activated sludge treatment technologies.

### Off-site emissions from discharge pathways

Treated wastewater was discharged in one of 10 different pathways. Table 5 shows emission factors of CO_2_, N_2_O and CH_4_ of each discharge pathway. The effluent emission factors of CH_4_ and N_2_O were adopted from IPCC 2019, while the CO_2_ emission factors of the treated effluent were derived from the appendix of IPCC 2019 (IPCC 2019, Volume 5, Chapter 6, Page 59-Page 60)^15^. The detailed derivation process of CO_2_ emission factor of effluent discharge refers to Supplementary Information ‘CO_2_ emission factor of effluent discharge’. Emissions from discharge pathways were calculated by Eq. 2.1–2.3:

21 $$\begin{array}{c}CH{4}_{eff,i}=E{F}_{eff,CH4,j}\ast CO{D}_{out,i}\ast 28\end{array}$$

22 $$\begin{array}{c}N2{O}_{eff,i}=E{F}_{eff,N2O,j}\ast T{N}_{out,i}\ast 265\end{array}$$

23 $$\begin{array}{c}CO{2}_{eff,i}=E{F}_{eff,CO2,j}\ast CO{D}_{out,i}\end{array}$$

where, $CH{4}_{eff,i}$, $N2{O}_{eff,i}$ and $CO{2}_{eff,i}$ are CH_4_, N_2_O and CO_2_ emissions (g CO_2_eq/year) from the discharge pathway *j* in the *i*th WWTP. $E{F}_{eff,CH4,j}$ (g CH_4_/kg COD effluent), $E{F}_{eff,N2O,j}$ (g N_2_O/kg TN effluent) and $E{F}_{eff,CO2,j}$ (g CO_2_/kg COD effluent) are effluent emission factors of the discharge pathway *j* of the *i*th WWTP. $CO{D}_{out,i}$ (kg COD effluent/year) and $T{N}_{out,i}$ (kg TN effluent/year) are annually effluent COD and TN mass of the *i*th WWTP.

### Off-site emissions from electricity consumption

The calculation of GHG emissions from electricity consumption is shown in Eq. 3.1. Baseline emission factors for regional power grids in China^41–44^ were used in this study. Only CO_2_ is considered for emission factors for regional power grids without considering N_2_O and CH_4_ due to their small contributions. China’s baseline emission factors for regional power grids are presented in Table 4.

31 $$\begin{array}{c}CO{2}_{ele,i}=E{F}_{ele,CO2,j}\ast El{e}_{con,i}\end{array}$$

where, $CO{2}_{ele,i}$ is the CO_2_ emission from electricity consumption (kg CO_2_/year). $E{F}_{ele,CO2,j}$ (kg CO_2_/kWh) denotes the CO_2_ emission factor of province *j* of the studied WWTP. $El{e}_{con,i}$ (kWh/year) refers to the electricity consumption of the *i*th WWTP.

**Table 4:** Baseline emission factors for regional power grid in China from 2006 to 2019 (Unit: kg CO_2_/kWh).

Regions in China	2006	2007	2008	2009	2010	2011	2012	2013	2014	2015	2016	2017	2018	2019
North	0.983	1.030	0.993	0.894	0.870	0.811	0.798	0.804	0.800	0.760	0.725	0.713	0.708	0.712
Northeast	1.005	1.050	1.030	0.927	0.910	0.842	0.852	0.862	0.841	0.780	0.780	0.720	0.678	0.661
East	0.864	0.905	0.884	0.783	0.769	0.749	0.757	0.761	0.748	0.703	0.678	0.648	0.589	0.590
Central	0.944	0.975	0.997	0.853	0.771	0.724	0.734	0.738	0.723	0.651	0.615	0.606	0.571	0.572
Northwest	0.841	0.850	0.877	0.834	0.841	0.793	0.766	0.742	0.705	0.631	0.639	0.619	0.643	0.666
South	0.778	0.843	0.880	0.788	0.713	0.632	0.657	0.650	0.678	0.630	0.587	0.542	0.503	0.509
Hainan	0.846	0.836	0.829	0.773	0.765	0.632	0.657	0.650	0.678	0.630	0.587	0.542	0.503	0.509

**Table 5:** Emission factors of different GHG emissions from discharge pathways.

		CH_4_	N_2_O	CO_2_
		(g CH_4_/kg COD effluent)	(g N_2_O/kg TN effluent)	(g CO_2_/kg COD effluent)
1	Discharge into seas directly	8.75	7.90	570.90
2	Discharge into rivers, lakes, reservoirs etc. directly	47.50	7.90	570.90
3	Enter sewers first, then discharge into rivers, lakes, and reservoirs	47.50	7.90	570.90
4	Enter sewers first, then discharge into seas	8.75	7.90	570.90
5	Enter municipal WWPTs	0.00	0.00	0.00
6	Discharge into sewage irrigated farmlands directly	0.00	8.00	—
7	Discharge into soil	0.00	8.00	—
8	Enter other facilities (decentralized wastewater treatment facilities)	0.00	0.00	0.00
9	Centralized industrial WWTPs	0.00	0.00	0.00
10	Other discharge pathways	27.50	7.90	570.90

Note: CH_4_ and N_2_O emission factors of discharge pathways of 5, 8, and 9 are zero, as they belong to the pathway of ‘flowing sewer’, and CH_4_ and N_2_O emission factors for the discharge pathway of ‘flowing sewer (open or closed)’ are zero in IPCC 2019. We also assumed that there was no CO_2_ generation under the pathway of ‘flowing sewer’. Discharge pathway 6 and 7 were regarded as discharge into soil in this study. From IPCC 2019, default CH_4_ emission factor of the pathway of discharge into soil was 0 g CH_4_/kg COD effluent. We did not consider CO_2_ emissions of discharge into soil, because of a lack of data on the CO_2_ emission factor of discharge into soil.

### Uncertainty analysis

The uncertainty of GHG emissions was mainly caused by emission factors. Since calculation of activity data of each WWTP was based on annual on-site monitored data of the volume of treated wastewater, influent and effluent concentration of pollutants and electricity consumption, there is no uncertainty for activity data. We analysed GHG emissions uncertainty induced by biological treatment processes and discharge pathways. The uncertainty caused by electricity consumption was not considered, because China’s regional power grid baseline emission factors are based on specific values rather than ranges.

For the emission factors of biological treatment processes, we acquired the minimum, maximum and average emission factors of each technology from the literature. Then, we used the following Eq. 4.1 and 4.2 to calculate the uncertainty of emission factors.

41 $$\begin{array}{c}Uncertainty\;lower\;bound\;Ulb=\frac{\left(E{F}_{min}-E{F}_{ave}\right)}{E{F}_{ave}}\ast 100 \% \end{array}$$

42 $$\begin{array}{c}Uncertainty\;upper\;bound\;Uub=\frac{\left(E{F}_{max}-E{F}_{ave}\right)}{E{F}_{ave}}\ast 100 \% \end{array}$$

Since the CH_4_ emission factor was determined by the multiplication of the maximum producing potential (B_0_) and the methane correction factor (MCF), its uncertainty was measured by Eq. 4.3. The uncertainty of B_0_ (*U*_*B*0_) is ± 30% in IPCC 2019, and the uncertainty of MCF (*U*_*MCF*_) was determined by Eq. 4.1 and 4.2. The uncertainties of N_2_O and CO_2_ emission factors of discharge pathways were calculated by Eq. 4.1 and 4.2.

43 $$\begin{array}{c}{U}_{CH4}=\pm \,\sqrt{{U}_{{B}_{0}}^{2}+{U}_{MCF}^{2}}\end{array}$$

We applied Monte Carlo simulations to analyse the combined uncertainty of emission factors and activity data. Emission factors of CH_4_, N_2_O and CO_2_ of biological treatment processes and discharge pathways all follow triangular distributions, because ‘upper and lower and a preferred value are provided (IPCC 2006, Volume 1, Chapter 3, Page 22)’^15^ in this study. Random sampling on emission factors was performed 100,000 times, then multiplied by activity data of each GHG in each WWTP, generating 100,000 values for GHG emissions. Finally, uncertainty ranges of 95% confidence intervals of GHG emissions were adopted.

Other causes that may induce uncertainties include ‘Measurement error’, ‘Lack of completeness’ and ‘Misreporting or misclassification’. With regard to the measurement error in a real WWTP, the measured influent and effluent concentration of pollutants and electricity consumption may be incorrect. But this uncertainty is difficult to quantify and control in this study. In terms of lack of completeness, the original data was incomplete for all WWTPs. For instance, data of some indicators was lacking, e.g., volume of treated wastewater, influent, or effluent concentrations of COD. When a WWTP does not have sufficient indicators, the WWTP was removed, and its emissions were not calculated. For the misreporting or misclassification, accurate classification of treatment technologies is the basis for calculating GHG emissions of secondary biological treatment processes, but uncertainties caused by misreporting and/or misclassification of treatment technologies are possible and cannot be easily rectified.

## Data Records

The dataset of “Greenhouse gas emissions of wastewater treatment plants in China from 2006 to 2019” is made public under Figshare^45^. There are 400,512 data records in the dataset. These include:

- 399,420 firm-level GHG emission inventories (57,060 firms, i.e., 57,060 WWTPs and other wastewater treatment facilities; for each firm, there are CH_4_, N_2_O and CO_2_ emissions from biological treatment processes, CO_2_ emissions from electricity consumption, and CH_4_, N_2_O and CO_2_ emissions from the discharge pathways);
- 70 annual biological treatment GHG emission inventories (from 2006–2019, CH_4_ and N_2_O emissions calculated by IPCC 2019 methodology, and CH_4_, N_2_O and CO_2_ emissions calculated as described in the section of Methods);
- 42 annual effluent GHG emission inventories (from 2006–2019, CH_4_ and N_2_O emissions calculated by the IPCC 2019 methodology, and CO_2_ emissions calculated by the method of this paper);
- 14 annual electricity CO_2_ emission inventories (from 2006–2019);
- 322 annual CO_2_eq emissions of different technologies from biological treatment processes (from 2006–2019, 23 technology categories);
- 322 are annual CO_2_ emissions of different technologies from electricity consumption (from 2006–2019, 23 technology categories);
- 322 annual CO_2_eq emissions of different technologies from discharge pathways (from 2006–2019, 23 technology categories).

In this study, the firm-level GHG emission inventory provides a foundation for the remaining emission inventories. Based on the firm-level GHG emission inventory, annual CH_4_, N_2_O and CO_2_ emission inventories of biological treatment processes, effluent and electricity consumption are presented, and annual total CO_2_eq emissions of different technologies from biological treatment processes, electricity consumption and discharge pathways are also quantified.

**Fig. 3:**
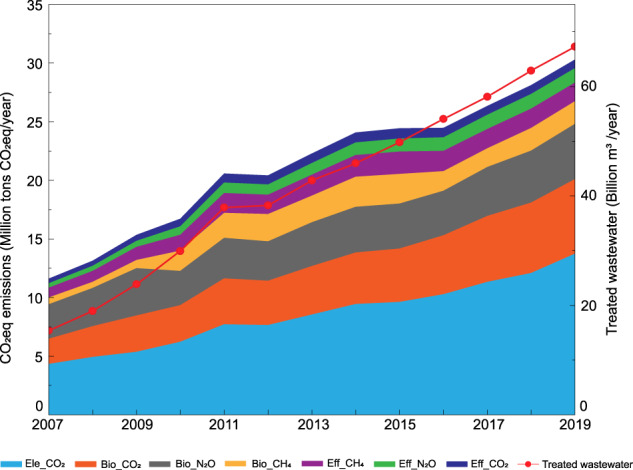
China’s GHG emissions from wastewater treatment (in million tons CO_2_eq) and treated wastewater (in billion cubic meters) 2006 to 2019. Ele, Bio and Eff indicate GHG emissions from electricity consumption, biological treatment processes and effluent discharge.

**Fig. 4:**
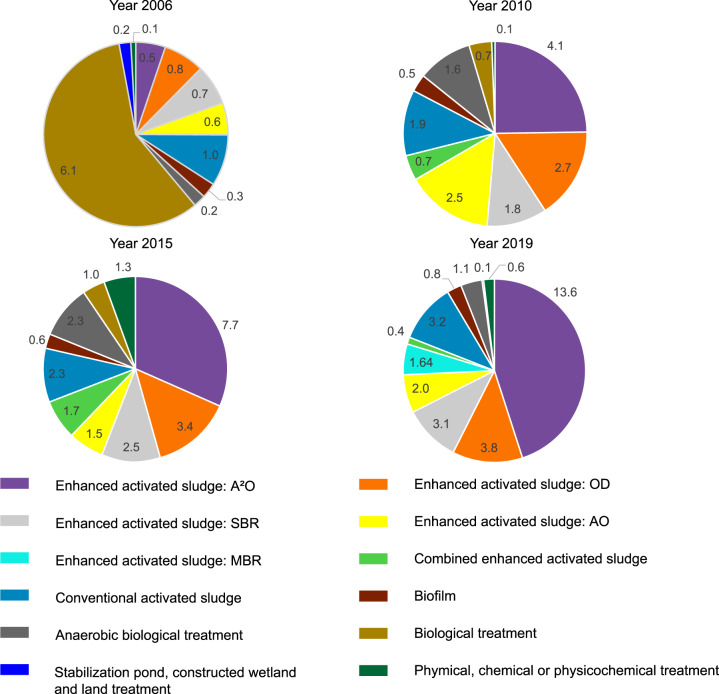
Structure of treatment technology in total CO_2_eq emissions in 2006, 2010, 2015 and 2019 (in million tons CO_2_eq). GHG emissions from enhanced activated sludge processes and conventional activated sludge accounted for a large proportion (>80%) in 2010, 2015 and 2019. While the percentage from biological treatment process was very high (58%) in 2006, because for some WWTPs, their subcategories of biological treatment processes were not reported in the original dataset. In this case, their treatment technologies were named as biological treatment, and their GHG emissions were estimated by emission factors of the process of activated sludge treatment in this study.

Figure 3 presents annual CH_4_, N_2_O and CO_2_ emissions from different emission sources and annual treated wastewater from 2006 to 2019. The pie charts in Fig. 4 show the structure of treatment technology in total CO_2_eq emissions in 2006, 2010, 2015 and 2019, respectively. Treatment technologies are classified by main categories of processes based on the classification in Table 1. Since the enhanced activated sludge process is the main wastewater treatment technology in China and it includes many sub-categories, the emission structure of sub-categories (i.e., AO, A^2^O, OD and SBR) of the enhanced activated sludge process is also shown in pie charts.

## Technical Validation

### Uncertainty analysis

#### Uncertainty of emission factors

The uncertainty of CH_4_, N_2_O and CO_2_ emission factors of biological treatment technologies is presented in Table 6. For comparison, we also list the uncertainty of CH_4_ and N_2_O emission factors based on IPCC 2019. The analysis by IPCC 2019 shows higher uncertainty in terms of CH_4_ and N_2_O emission factors of a majority of biological treatment technologies, due to its less detailed classification of technologies. For instance, different activated sludge technologies in IPCC 2019 possess the same emission factors and uncertainties, because IPCC 2019 classifies all activated sludge processes into one aerobic process category. However, processes of AO, A^2^O, SBR and OD are quite different, although they are all activated sludge technologies. Since we collected GHG emission factors based on different categories of traditional or enhanced activated sludge processes, emission factors and their uncertainties of processes of AO, A^2^O, SBR and OD are different and have different ranges (Table 6). However, on-site emission factors of certain processes are rarely reported in the literature, and we cannot obtain their emission factors based on detailed process classification. For example, we applied a CH_4_ emission factor (200 g CH_4_/kg COD) of the anaerobic process from IPCC 2019 to four different anaerobic processes (i.e., anaerobic hydrolysis, typical anaerobic reactors, anaerobic biofilter, and other anaerobic biological treatment), due to a lack of their on-site emission factors from references. Therefore, reported uncertainties (−30%,39%) for CH_4_ emission factors of the four anaerobic processes are the same. Overall, the uncertainties of GHG emission factors of different biological treatment technologies were relatively high. One of the main reasons is that GHG emission factors are strongly affected by different operational parameters^46–49^ (temperature, pH, dissolved oxygen (DO), sludge retention time (SRT), hydraulic retention time (HRT), influent chemical oxygen demand (COD) to total nitrogen ratio (C/N), influent chemical oxygen demand (COD) to total phosphorus ratio (C/P), etc.) of these WWTPs.

**Table 6:** Uncertainty of CH_4_, N_2_O and CO_2_ emission factors of biological treatment technologies.

	IPCC 2019		IPCC 2019		This study		This study		This study	
	CH_4_		N_2_O		CH_4_		N_2_O		CO_2_	
Aerobic Biological Treatment	−95%	202%	−99%	184%	−43%	57%	−83%	158%	−4%	0%
Activated Sludge	−95%	202%	−99%	184%	−43%	57%	−83%	158%	−4%	0%
AO	−95%	202%	−99%	184%	−68%	351%	−13%	15%	−86%	86%
A^2^O	−95%	202%	−99%	184%	−93%	324%	−97%	456%	−57%	54%
OD	−95%	202%	−99%	184%	−33%	22%	−50%	228%	−71%	49%
SBR	−95%	202%	−99%	184%	−86%	28%	−40%	50%	−82%	82%
AB	−95%	202%	−99%	184%	−43%	57%	−83%	158%	−4%	0%
Biofilm	—	—	−99%	184%	—	—	−99%	119%	0%	120%
Biofilter	—	—	−99%	184%	—	—	−99%	119%	0%	120%
Rotating Biological Contactor	—	—	−99%	184%	—	—	−99%	119%	0%	120%
Biological Contact Oxidation	—	—	−99%	184%	—	—	−99%	119%	0%	120%
Anaerobic Biological Treatment	−30%	39%	—	—	−30%	39%	—	—	−30%	30%
Anaerobic Hydrolysis	−30%	39%	—	—	−30%	39%	—	—	−30%	30%
Typical Anaerobic Reactors	−30%	39%	—	—	−30%	39%	—	—	−30%	30%
Anaerobic Biofilter	−30%	39%	—	—	−30%	39%	—	—	−30%	30%
Other Anaerobic Biological Treatment	−30%	39%	—	—	−30%	39%	—	—	−30%	30%
Stabilization Pond, Constructed Wetland and Land Treatment	−54%	58%	−97%	173%	−54%	58%	−97%	173%	−17%	14%
Stabilization Lagoon	−47%	52%	−99%	186%	−47%	52%	−99%	186%	−9%	6%
Oxidation Lagoon	−95%	202%	−99%	184%	−95%	202%	−99%	184%	−4%	0%
Anaerobic Lagoon	−30%	39%	—	—	−30%	39%	—	—	−30%	30%
Facultative Lagoon	−100%	58%	−99%	184%	−100%	58%	−99%	184%	−4%	0%
Aerated Lagoon	−95%	202%	−99%	184%	−95%	202%	−99%	184%	−4%	0%
Constructed Wetland	−77%	73%	−80%	80%	−77%	73%	−80%	80%	−30%	30%
Subsurface Flow Constructed Wetland	−45%	45%	−79%	79%	−45%	45%	−79%	79%	−30%	30%
Surface Flow Constructed Wetland	−85%	81%	−90%	90%	−85%	81%	−90%	90%	−30%	30%
Land Infiltration	−39%	53%	−100%	129%	−39%	53%	−100%	129%	−17%	14%
Biological Treatment	−95%	202%	−99%	184%	−95%	202%	−99%	184%	−4%	0%

Note: The symbol ‘—’ indicates the uncertainty of CH_4_, N_2_O or CO_2_ emission factor of a biological treatment technology is not existed when the default emission factor of a treatment process is zero.

The uncertainty of CH_4_, N_2_O and CO_2_ emission factors of 10 discharge pathways is shown in Table 7. Since CH_4_ and N_2_O emission factors for the discharge pathway of ‘flowing sewer (open or closed)’ are zero in IPCC 2019, we assumed that there was no CO_2_ generation under this flowing condition. We regarded discharge pathways via municipal WWPTs, centralized industrial WWTPs and other facilities (decentralized wastewater treatment facilities) as discharge pathway of ‘flowing sewer’. Therefore, we do not report any uncertainty of CH_4_, N_2_O and CO_2_ emission factors of entering municipal WWTPs, industrial WWTPs and other facilities. We considered the discharge pathway of ‘other discharge pathways’ in this study as ‘discharge to aquatic environments (Tier 1)’ in IPCC 2019, and its uncertainties of CH_4_ (−100%, 148%) and N_2_O emission factors (−90%, 1394%) are the largest compared with other discharge pathways. Because there are very few studies on the CO_2_ emission factor of the treated effluent, we derived CO_2_ emission factors of lakes, rivers and reservoirs from the appendix of IPCC 2019 (IPCC 2019, Volume 5, Chapter 6, Page 59-Page 60)^15^, and we assumed that pathways of discharging into sea and ‘others’ also have the same CO_2_ emission factors. Thus, their CO_2_ emission factor uncertainties were all the same, with the uncertainty of (−12%, 20%).

**Table 7:** Uncertainty of emission factors of different discharge pathways.

		CH_4_		N_2_O		CO_2_	
1	Direct discharge into seas	−94%	80%	−90%	1394%	−12%	20%
2	Direct discharge into rivers, lakes, reservoirs etc.	−65%	52%	−90%	1394%	−12%	20%
3	Enter sewers first, then discharge into rivers, lakes, and reservoirs	−65%	52%	−90%	1394%	−12%	20%
4	Enter sewers first, then discharge into seas	−94%	80%	−90%	1394%	−12%	20%
5	Enter municipal WWPTs	—	—	—	—	—	—
6	Direct discharge into sewage irrigated farmland			100%	116%		
7	Discharge into soil	—	—	100%	116%	—	—
8	Enter other facilities (decentralized wastewater treatment facilities)	—	—	—	—	—	—
9	Enter centralized industrial WWTPs	—	—	—	—	—	—
10	Other discharge pathways	−100%	148%	−90%	1394%	−12%	20%

Note: The symbol ‘-’ indicates the uncertainty of CH_4_, N_2_O or CO_2_ emission factor of a discharge pathway is not existed when the default emission factor of a discharge pathway is zero or is not existed.

#### Combined uncertainty of GHG emissions

**Fig. 5:**
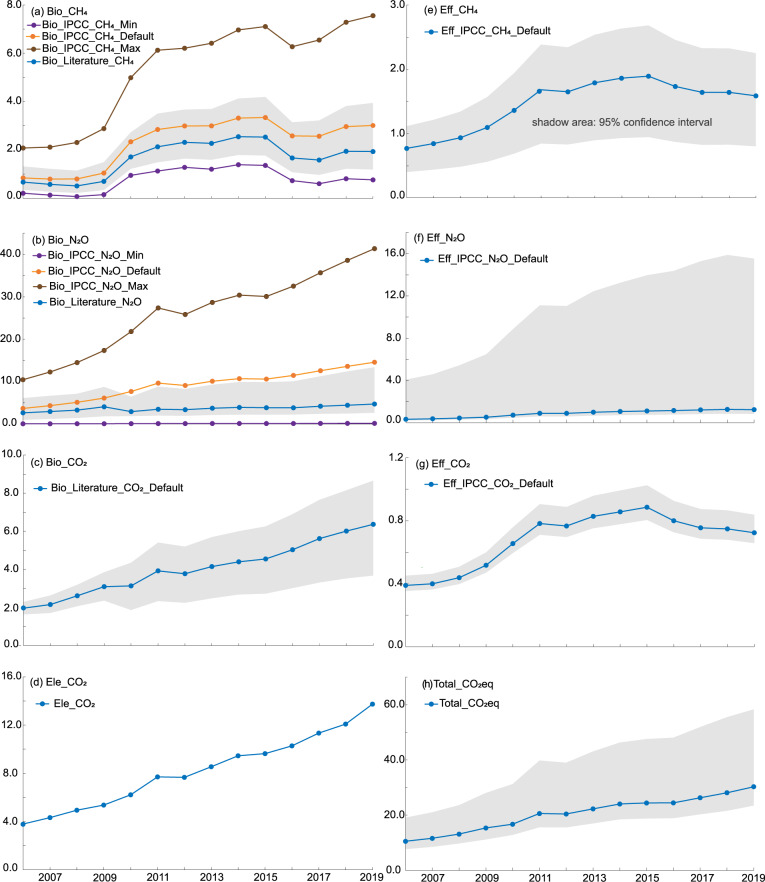
Trend and uncertainty of GHG emissions from WWTPs in China from 2006 to 2019 (in million tons CO_2_eq). (**a**) Trend and uncertainty of CH_4_ emissions from biological treatment. (**b**) Trend and uncertainty of N_2_O emissions from biological treatment. (**c**) Trend and uncertainty of CO_2_ emissions from biological treatment. (**d**) CO_2_ emissions from electricity consumption. (**e**) Trend and uncertainty of CH_4_ emissions from effluent. (**f**) Trend and uncertainty of N_2_O emissions from effluent. (**g**) Trend and uncertainty of CO_2_ emissions from effluent. (**h**) Trend and uncertainty of total CO_2_eq emissions. Bio: biologocal. Eff: effluent. Ele: electricity. The shadow areas indicate the 95% confidence interval of GHG emissions. The uncertainty of electricity consumption is not shown in (**d**) because of unavailable uncetainty of power grid baseline emission factors.

The combined uncertainty of GHG emissions of biological treatment processes is presented in Table 8 and Fig. 5(a–c). The shadow areas shown in Fig. 5 indicate the 95% confidence interval of GHG emissions. For comparison, CH_4_ and N_2_O emissions calculated by emission factors of IPCC 2019 are also shown in Fig. 5(a,b). From 2006 to 2019, the uncertainties of CH_4_, N_2_O and CO_2_ emissions in this study were (−57%, 124%), (−63%, 184%) and (−43%, 38%), respectively. But uncertainties of CH_4_ and N_2_O emissions calculated by the methodology of IPCC 2019 were (−91%, 189%) and (−99%, 184%). The minimum and maximum CH_4_ and N_2_O emissions calculated by IPCC 2019 were all outside of the shadow areas in Fig. 5(a,b), reflecting larger uncertainties than in our study.

**Table 8:** The combined uncertainty of GHG emissions from biological treatment.

	CH_4_ emissions				N_2_O emissions				CO_2_ emissions	
	This study		IPCC 2019		This study		IPCC 2019		This study	
2006	−48%	98%	−75%	147%	−63%	131%	−99%	184%	−18%	16%
2007	−52%	111%	−83%	166%	−60%	126%	−99%	184%	−22%	21%
2008	−57%	124%	−91%	189%	−57%	118%	−99%	184%	−22%	21%
2009	−52%	113%	−85%	176%	−55%	116%	−99%	184%	−24%	24%
2010	−29%	59%	−59%	113%	−37%	121%	−99%	184%	−41%	38%
2011	−30%	64%	−60%	115%	−43%	154%	−99%	184%	−41%	37%
2012	−29%	59%	−57%	108%	−43%	146%	−99%	184%	−41%	37%
2013	−30%	62%	−60%	114%	−43%	149%	−99%	184%	−41%	37%
2014	−30%	62%	−58%	110%	−44%	154%	−99%	184%	−39%	36%
2015	−30%	65%	−59%	113%	−43%	157%	−99%	184%	−41%	37%
2016	−35%	88%	−72%	143%	−42%	164%	−99%	184%	−40%	37%
2017	−39%	103%	−76%	155%	−43%	169%	−99%	184%	−41%	36%
2018	−37%	96%	−73%	146%	−44%	180%	−99%	184%	−42%	35%
2019	−39%	104%	−75%	151%	−44%	184%	−99%	184%	−43%	36%

The combined uncertainty of effluent GHG emissions is presented in Table 9 and Fig. 5(e–g). The overall uncertainties of the effluent N_2_O were very high (−33%, 1161%), mainly resulting from high uncertainty of the effluent N_2_O emission factor (−100%, 1394%). N_2_O emission factors vary substantially between WWTPs, due to different process designs and operational conditions^46,47^. Effluent CH_4_ and CO_2_ emission uncertainties were relatively low, with values of (−52%, 29%) and (−9%, 16%), respectively. The uncertainty of total GHG emissions of WWTPs are shown in Fig. 5(h) and Table S4. The uncertainties of total GHG emissions from WWTPs were about (−27%, 97%).

**Table 9:** The combined uncertainty of GHG emissions from effluent.

	CH_4_ emissions		N_2_O emissions		CO_2_ emissions	
2006	−52%	29%	−33%	1160%	−9%	16%
2007	−52%	29%	−33%	1151%	−9%	16%
2008	−52%	29%	−33%	1148%	−9%	16%
2009	−52%	29%	−33%	1152%	−9%	16%
2010	−52%	29%	−33%	1148%	−9%	16%
2011	−52%	28%	−33%	1149%	−9%	16%
2012	−52%	28%	−33%	1149%	−9%	16%
2013	−52%	28%	−33%	1159%	−9%	16%
2014	−52%	28%	−33%	1150%	−9%	16%
2015	−52%	28%	−33%	1158%	−9%	16%
2016	−52%	28%	−33%	1145%	−9%	16%
2017	−52%	28%	−33%	1159%	−9%	16%
2018	−52%	28%	−33%	1160%	−9%	16%
2019	−52%	28%	−33%	1161%	−9%	16%

### Comparison with existing estimations

Several studies on CH_4_ or N_2_O emissions of WWTPs at the national level in China have been reported^7–13^. In Table S5, we list wastewater GHG estimations in the literature for comparison. In most cases, the current estimation results are not comparable. The use of different system boundaries across studies is one of the main reasons. For instance, CH_4_ emissions (76.2 Mt CO_2_eq) of wastewater from China’s second biennial update report on climate change^50^ in 2014 refer to emissions from both industrial and domestic wastewater at the national level and activity data was obtained from the Environmental Statistics Yearbook, while *Zhao et al*.^10^ considered CH_4_ emissions (29.2 Mt CO_2_eq) from 2019 WWTPs at the firm level in 229 cities in 2014 and the data was from the Urban Drainage Statistic Yearbook. Their results are not comparable, since 2019 WWTPs in *Zhao et al*.’s study contained mainly prefecture-level municipal WWTPs but excluded county-level and industrial WWTPs in China, and it is not clear how many WWTPs/wastewater treatment facilities are included in China’s second biennial update report. Therefore, the activity data and CH_4_ emissions were not comparable in these two studies, although they all used IPCC 2006 method for their inventories. In our paper, on-site CH_4_ emissions from 4455 WWTPs and 718 other treatment facilities were estimated to be 2.55 Mt CO_2_eq in 2014, which were about one tenth of *Zhao et al.’*s results. This discrepancy was caused by using different system boundaries and the use of different emission factors.

Most studies used emission factors from the IPCC, but even CH_4_ emission factors from IPCC 2006 and IPCC 2019 are quite different. The default methane correct factor (MCF) in IPCC 2019 is 0.03, while this value is 0.3 in IPCC 2006 for overloaded WWTPs, and it may differ by one order of magnitude for CH_4_ emissions. Our uncertainty analysis shows that CH_4_ emissions calculated by IPCC 2019 are about 20%–62% larger than our research, and uncertainties caused by IPCC 2019 were much higher than in this study. In other cases, emission factors from the literature without distinguishing different technologies were used to estimate GHG emissions. For example, the MCF of 0.165 was used to calculate CH_4_ emissions induced by domestic wastewater in several studies^7–9,12^. By using MCF 0.165, CH_4_ emissions from domestic wastewater were around 28 Mt CO_2_eq in 2014 in *Du et al*.^7^ While *Yan et al*.^11^ obtained that the estimated CH_4_ emissions were 0.77 Mt CO_2_eq in 2014 by using the emission factor of 2.3064 kg CH_4_/t COD removed. The discrepancy of CH_4_ estimations between *Du et al*.^7^ and *Yan et al*.^11^ in 2014 was nearly 36 times. In comparison, estimated CH_4_ emissions in our study are 2.6 Mt CO_2_eq. Comparing *Guo et al*.^13^ with our study, the main difference was that *Guo et al*. applied only one N_2_O emission factor (0.035 kg N_2_O-N/kg TN) to all treatment technologies and their CH_4_ emission factors were based on different provinces^51^. But our CH_4_ and N_2_O emission factors were based on the specific technology of each WWTP. Total CH_4_ and N_2_O emissions from biological wastewater treatment and CO_2_ emissions from electricity consumption in *Guo et al*. in 2016 were 31.4 Mt CO_2_eq, which are about twice of our result (15.9 Mt CO_2_eq).

Misuse of CH_4_ emission calculation formula in IPCC 2006 or IPCC 2019 is another reason leading to incomparability of CH_4_ emissions. Normally, CH_4_ emissions are equal to a CH_4_ emission factor times the difference between total influent COD (or BOD) mass and COD (or BOD) removed in the form of sludge. Total influent COD (or BOD) mass minus COD (or BOD) removed in the form of sludge means that organic components transferred to sludge do not generate direct CH_4_, but only the remaining organic matter in the wastewater has potential to emit CH_4_. Therefore, the unit (kg CH_4_/kg BOD or kg CH_4_/kg COD) of CH_4_ emission factor in IPCC indicates CH_4_ emissions per unit remaining organic mass in the influent after considering COD (or BOD) transferred to the sludge, rather than CH_4_ emissions per unit influent COD (or influent BOD) or per unit COD (or BOD) removed^9^. In addition, organic matter removed in the form of sludge was assumed as being zero for all treatment technologies^7–10,12^. The reasons for the incorrect assumption may be the lack of data on sludge generation, and the method to estimate organic components removed in the form of sludge is not mentioned in IPCC 2006, or the lack of background on wastewater treatment. The assumption may overestimate CH_4_ emissions as most aerobic biological treatment technologies generate sludge during wastewater treatment. However, IPCC 2019 updated the method to account CH_4_ emissions based on IPCC 2006, especially providing equations and detailed information to estimate COD (or BOD) transferred to sludge, which provides guidance for accurate CH_4_ accounting.

### Limitations

We have four main limitations in this study. (1) A WWTP may have one or more wastewater treatment streams, and for each treatment stream, it may contain primary, secondary or tertiary treatment processes, while for each process (normally for a secondary treatment process), it has multiple treatment technologies. But to simplify GHG emissions estimation of biological treatment technologies of the secondary treatment process of a WWTP, the decision tree (Fig. 2) was applied to determine the main category of treatment technology and its corresponding emission factors, especially when a WWTP has several secondary treatment technologies. (2) Our emission factors of different biological treatment technologies were not based on the monitoring of each wastewater treatment plant. But we used emission factors in line with Chinese conditions. The emission factors were acquired from different references, such as on-site monitoring of specific biological technologies or modelling estimations in the literature, which was based on case studies of WWTPs in China. However, emission factors of some biological technologies, such as CH_4_ and CO_2_ emission factors of anaerobic technologies and constructed wetlands, were missing for China, thus we used IPCC emission factors for these technologies instead. On the other hand, given that emission factors of a specific biological treatment technology are greatly affected by operational conditions, different WWTPs with the same biological technology may have different emission factors. Therefore, GHG emission factors of a biological technology obtained from references are not representative for real emission factors of all WWTPs with the same technology. (3) GHG emissions from industrial WWTPs are not available and thus not included in our study although being important GHG emission sources of wastewater treatment systems^52–54^. For instance, *Xing et al*. reported that CH_4_ emissions from on-site industrial wastewater treatment were always higher than that of domestic wastewater treatment between 2003 and 2008 in China. CH_4_ emissions from industrial and domestic wastewater treatment were 0.95 Mt and 0.91 Mt respectively in 2008^54^. (4) Anthropogenic CO_2_ emissions (or fossil CO_2_ emissions) from biological treatment processes and discharge pathways are of main concern compared with biogenic CO_2_ emissions, but we did not calculate fossil CO_2_ emissions separately, because the CO_2_ emission factors available in the literature are only reported as total CO_2_, rather than separate fossil and biogenic CO_2_.

## Supplementary information

Supporting Information

## Data Availability

The scripts used to calculate firm level GHG emissions of wastewater treatment facilities are available in the Zenodo repository: 10.5281/zenodo.6052815^55^.
