# Supplementary material for: Greenhouse gas emissions from municipal wastewater treatment facilities in China from 2006 to 2019
Source: Sci Data. 2022 Jun 16;9:317. doi: 10.1038/s41597-022-01439-7 (PMC9203788; doi:10.1038/s41597-022-01439-7)
Supplement: Supplementary file 1 — Supporting Information [file 41597_2022_1439_MOESM1_ESM.docx]

# Supporting Information Appendix

# Greenhouse gas emissions from municipal wastewater treatment facilities in China from 2006 to 2019

Dan Wang^1,7^ Weili Ye^2,7^ Guangxue Wu^3^, Ruoqi Li^1,4^ Yuru Guan^1^, Wei Zhang^2,5,*^ Junxia Wang^6^, Yuli Shan^1^, Klaus Hubacek^1,*^

1. Integrated Research on Energy Environment and Society (IREES), Energy Sustainability Research Institute Groningen (ESRIG), University of Groningen, Groningen 9747 AG, The Netherlands

2. The Center for Beijing-Tianjin-Hebei Regional Environment and Ecology, Chinese Academy of Environmental Planning, Beijing 100012, China

3. Civil Engineering, School of Engineering, College of Science and Engineering, National University of Ireland, Galway, Galway H91 TK33, Ireland

4. State Key Laboratory of Pollution Control and Resource Reuse, School of the Environment, Nanjing University, Nanjing 210023, China

5. State Environmental Protection Key Laboratory of Environmental Planning and Policy Simulation, Chinese Academy of Environmental Planning, Beijing 100012, China

6. State Environmental Protection Key Laboratory of Quality Control in Environmental Monitoring, China National Environmental Monitoring Centre, Beijing 100012, China

7. These authors contributed equally to this work.

Correspondence emails: [zhangwei@caep.org.cn](mailto:zhangwei@caep.org.cn) (W.Z.); [k.hubacek@rug.nl](mailto:k.hubacek@rug.nl) (K.H.)

**This PDF file includes**

Table S1 Summary of CH_4_ emission factors of biological treatment processes from the literature

Table S2 Summary of N_2_O emission factors of biological treatment processes from the literature

Table S3 Summary of CO_2_ emission factors of biological treatment processes from the literature

Table S4 The uncertainty of total GHG emissions of WWTPs

Table S5 Comparing wastewater GHG estimations in this study with existing research

Derivation process of CO_2_ emission factor of effluent discharge

# Supplementary Tables

**Table S1 Summary of CH_4_ emission factors of biological treatment processes from the literature**

| References | Processes | EF in this study | Unit in this study | EF in the literature | Unit in the literature | Unit conversion information or other notes |
| --- | --- | --- | --- | --- | --- | --- |
| (Aboobakar et al., 2013)^1^ | Activated sludge | 0.4-1.1 (0.7) | g CH_4_/ kg COD removed | 0.4-1.1 (0.7) | g CH_4_/ kg COD removed | * A full-scale nitrifying activated sludge plant in UK (just monitored CH_4_ secondary process); No sludge related sampling location |
| (Ren et al., 2013)^2^ | reversed A^2^O | 0.84 | g CH_4_/kg COD removed | 0.982 | g CH_4_/m^3^ wastewater | * Average COD influent and effluent.  * COD influent: 1188.4 mg/L.  * COD effluent: 20.4 mg/L |
| (Yan et al., 2014)^3^ | reversed A^2^O | 0.7–2.4 (1.4) | g CH_4_/kg COD removed | 0.7–2.4 (1.4) | g CH_4_/kg COD removed | * Beijing, China; On-site measurement; full scale WWTP emission factor; No sludge related sampling point |
| (Liu et al., 2014)^4^ | A^2^O | 0.189-1.29 (0.52) | g CH_4_/kg COD removed | 0.066-0.452 (0.182) | g CH_4_/ m^3^ wastewater | * Average COD influent and effluent  * COD influent: 400 mg/L  * COD effluent: 50 mg/L  * Beijing, China; On-site measurement; full scale WWTP emission factor * No sludge related sampling location |
| (Wang et al., 2011)^5^ | A^2^O | 0.86-1.35 (1.03) | g CH_4_/kg COD removed | 0.129-0.203 (0.155) | g CH_4_/ m^3^ wastewater | * COD average influent: 200 mg/L * COD effluent < 50 mg/L  * To estimate emission factor, COD average effluent was regarded as 50 mg/L  * Jinan, China; monitor every processing unit; EF was calculated based on whole process.  * Sampling location include sludge concentration tanks, sludge screw conveyor and sludge drying ground |
| (Yan et al., 2014)^3^ | A^2^O | 0.5–1.2 (0.9) | g CH_4_/kg COD removed | 0.5–1.2 (0.9) | g CH_4_/kg COD removed | * Beijing, China; On-site measurement; full scale WWTP emission factor; No sludge related sampling point |
| (Ren et al., 2013)^2^ | A^2^O | 11.28 | g CH_4_/kg COD removed | 5.22 | g CH_4_/ m^3^ wastewater | * Average COD influent and effluent  * COD influent: 497.3 mg/L  * COD effluent: 34.6 mg/L |
| (Liu et al., 2014)^4^ | SBR | 0.241-2.233 (1.252) | g CH_4_/kg COD removed | 0.088-0.815 (0.457) | g CH_4_/ m^3^ wastewater | * Average COD influent and effluent  * COD influent: 500 mg/L  * COD effluent: 35 mg/L  * Beijing, China; On-site measurement; full scale WWTP emission factor  * No sludge related sampling location |
| (Bao et al., 2016)^6^ | SBR | 2.26 | g CH_4_/kg COD removed | 0.81 | g CH_4_/ m^3^ wastewater | * The COD and total nitrogen removal efficiencies were higher than 90% and 50% respectively in both the AO and SBR WWTPs  * SBR wastewater inflow 75.2* 10^3^m^3^/d  * COD influent was 397.7 mg/L  * TN influent was 62.0 mg/L  * Beijing, China; On-site measurement; full-scale emission factor; No sludge related sampling point |
| (Yan et al., 2014)^3^ | OD | 2.85–3.95 (3.3) | g CH_4_/kg COD removed | 2.85–3.95 (3.3) | g CH_4_/kg COD removed | * Beijing, China; On-site measurement; full scale WWTP emission factor; Sludge related sampling point: sludge thickener and sludge storage area |
| (Ren et al., 2013)^2^ | OD | 5.23 | g CH_4_/kg COD removed | 0.588 | g CH_4_/m^3^ wastewater | * Average COD influent and effluent * COD influent: 140.8 mg/L * COD effluent: 28.4 mg/L |
| (Bao et al., 2016)^6^ | AO | 0.24-3.34 (0.74) | g CH_4_/kg COD removed | 0.08-1.1 (0.24) | g CH_4_/ m^3^ wastewater | * The COD and total nitrogen removal efficiencies were higher than 90% and 50% respectively in both the AO and SBR WWTPs  * COD influent was 364.8 mg/L  * Data is derived from Fig.4 (b)  * Beijing, China; On-site measurement; full-scale emission factor; No sludge related sampling point |

Note: the values in brackets indicate the average of emission factors

**Table S2 Summary of N_2_O emission factors of biological treatment processes from the literature**

| References | Processes | EF in this paper | Unit in this paper | EF in the literature | Unit in the literature | Unit conversion information or other notes |
| --- | --- | --- | --- | --- | --- | --- |
| (Czepiel et al., 1995)^7^ | Activated sludge | 0.35-0.5 | g N_2_O/kg TN influent | 0.35-0.5 | g N_2_O/kg TN influent | - |
| (Brotto et al., 2015)^8^ | Activated sludge | 0.2-3.1 (1.2) | g N_2_O/kg TN influent | 0.2-3.1 (1.2) | g N_2_O/kg TN influent | - |
| (Yan et al., 2014)^3^ | Reversed A^2^O | 0.2-4.96 (1.23) | g N_2_O/kg TN influent | 0.02-0.2 (0.07) | g N_2_O/m^3^ wastewater | * Beijing, China; On-site measurement; full scale WWTP emission factor; No sludge related sampling point  * Reversed A^2^O: TN influent: 40.3 mg/L-98.8 mg/L  * Maximum N_2_O EF was estimated: 0.2/40.3*1000=4.96 g N_2_O/kg TN influent  * Minimum N_2_O EF was estimated: 0.02/98.8*1000= 0.2 g N_2_O/kg TN influent  * Preferred N_2_O EF was estimated: (0.07/40.3*1000+0.07/98.8*1000)/2 = (1.74+0.71)/2= 1.23 g N_2_O/kg TN influent |
| (Ren et al., 2013)^2^ | Reversed A^2^O | 1.12 | g N_2_O/kg TN influent | 0.128 | g N_2_O/m^3^ wastewater | * TN average influent: 114.4 mg/L  * Monitor every processing unit; sampling position include sludge concentration tanks; sludge screw conveyor; sludge drying ground  * A full-scale WWTP in Qingdao in China (secondary process: reverse A^2^O)  * EF was calculated based on whole process |
| (Sun et al., 2013)^9^ | A^2^O | 19.5 | g N_2_O/kg TN influent | 19.5 | g N_2_O/kg TN influent | * No sludge related sampling location; Beijing, China; EF was calculated based on whole process |
| (Yan et al., 2014)^3^ | A^2^O | 0.3-1.49 (0.88) | g N_2_O/kg TN influent | 0.03-0.06 (0.05) | g N_2_O/m^3^ wastewater | * Beijing, China; On-site measurement; full scale WWTP emission factor; No sludge related sampling point  * A^2^O: TN influent: 40.3 mg/L-98.8 mg/L  * Maximum N_2_O EF was estimated: 0.06/40.3*1000= 1.49 g N_2_O/kg TN influent  * Minimum N_2_O EF was estimated: 0.03/98.8*1000= 0.3 g N_2_O/kg TN influent  * Preferred N_2_O EF was estimated: (0.05/40.3*1000+0.05/98.8*1000)/2 = (1.24+0.51)/2=0.88 g N_2_O/kg TN influent |
| (Wang et al., 2011)^10^ | A^2^O | 0.96-1.2 | g N_2_O /kg TN influent | 0.024-0.03 | g N_2_O/m^3^ wastewater | * TN average influent: 25 mg/L  * Jinan, China  * Monitor every processing unit; sludge related sampling position include high density settler tanks, high efficiency fiber filter beds, sludge concentration tanks |
| (Ren et al., 2013)^2^ | A^2^O | 1.52 | g N_2_O /kg TN influent | 0.122 | g N_2_O/m^3^ wastewater | * TN average influent: 80.1 mg/L  monitor every processing unit; sampling position include sludge concentration tanks; sludge screw conveyor; sludge drying ground  * a full-scale WWTP in Qingdao in China (secondary process: A^2^O)  * EF was calculated based on whole process |
| (Wang et al., 2016)^11^ | A^2^O | 0.95-34.4 (12.9) | g N_2_O /kg TN influent | 0.95-34.4 (12.9) | g N_2_O/kg TN influent | * Shanghai, China; one year  * EF was calculated based on only A^2^O process; other processes were not included |
| (Sun et al., 2013)^9^ | SBR | 65.2 | g N_2_O /kg TN influent | 65.2 | g N_2_O/kg TN influent | * No sludge related sampling location; Beijing, China; EF was calculated based on whole process |
| (Sun et al., 2013)^12^ | SBR | 56 | g N_2_O /kg TN influent | 56 | g N_2_O/kg TN influent | * 5.6% of N-inﬂuent was transformed into N_2_O and emitted (it indicates EF is 56g N_2_O-N/TN influent); * On-site measurement; full-scale emission factor; No sludge related sampling location; Beijing, China |
| (Bao et al., 2016)^6^ | SBR | 26.3 | g N_2_O /kg TN influent | 1.63 | g N_2_O/m^3^ wastewater | * The COD and total nitrogen removal efficiencies were higher than 90% and 50% respectively in both the AO and SBR WWTPs  * SBR influent wastewater inflow 75.2* 10^3^ m^3^/d  * COD influent was 397.7 mg/L  * TN influent was 62.0 mg/L  * Beijing, China; On-site measurement; full-scale emission factor; No sludge related sampling point |
| (Sun et al., 2015)^13^ | SBR | 26.9 | g N_2_O /kg TN influent | 26.9 | g N_2_O/kg TN influent | * Beijing, China; On-site measurement; full-scale emission factor; No sludge related sampling location |
| (Ren et al., 2013)^2^ | OD | 1.41 | g N_2_O /kg TN influent | 0.0773 | g N_2_O/m^3^ wastewater | * TN average influent: 54.7 mg/L  * Monitor every processing unit; sampling position include sludge concentration tanks; sludge screw conveyor; sludge drying ground  * A full-scale WWTP in Jinan in China (secondary process: OD)  * EF was calculated based on whole process |
| (Yan et al., 2014)^3^ | OD | 1.1-7.14 (2.89) | g N_2_O/kg TN influent | 0.1–0.4 (0.2) | g N_2_O/m^3^ wastewater | * Beijing, China; On-site measurement; full scale WWTP emission factor; Sludge related sampling point: sludge thickener and sludge storage area  * OD: TN influent: 56.0 mg/L-90.8 mg/L  * Maximum N_2_O EF was estimated: 0.4/56.0*1000= 7.14 g N_2_O /kg TN influent  * Minimum N_2_O EF was estimated: 0.1/90.8*1000= 1.1 g N_2_O /kg TN influent  * Preferred N_2_O EF was estimated: (0.2/56*1000+0.2/90.8*1000)/2 = (3.57+2.2)/2=2.89 |
| (Sun et al., 2015)^13^ | OD | 2.5 | g N_2_O/kg TN influent | 2.5 | g N_2_O/kg TN influent | * China, city is not clear; On-site measurement; full-scale emission factor; Sampling location include sludge distribution and concentration tank |
| (Bao et al., 2016)^6^ | AO | 12.12 | g N_2_O/kg TN influent | 0.6 | g N_2_O/m^3^ wastewater | * The COD and total nitrogen removal efficiencies were higher than 90% and 50% respectively in both the AO and SBR WWTPs  * AO wastewater inflow 443.5 * 10^3^m^3^/d  * COD influent was 364.8 mg/L  * TN influent was 49.5 mg/L  * Beijing, China; On-site measurement; full-scale emission factor; No sludge related sampling point |
| (Sun et al., 2015)^13^ | AO | 13.7 | g N_2_O/kg TN influent | 13.7 | g N_2_O/kg TN influent | * China, city is not clear; On-site measurement; full-scale emission factor; No sludge related sampling location |
| (Sun et al., 2017)^14^ | AO | 16 | g N_2_O/kg TN influent | 16 | g N_2_O/kg TN influent | * Beijing, China; On-site measurement; full-scale emission factor; No sludge related sampling point |
| (Wang et al., 2016)^15^ | BAF (biological aerated filter) | 0.17-12.61 | g N_2_O/kg TN influent | 0.17-12.61 | g N_2_O/kg TN influent | * A full-scale BAF were studied online for the first time  * The full scale WWTP include both A^2^O and BAF, but N_2_O was just monitored in BAF  * No sludge sampling point |
| (Lo et al., 2010)^16^ | Biofilm reactor | 5 | g N_2_O/kg TN influent | 5 | g N_2_O/kg TN influent | * Laboratory-scale  * The laboratory experiment was conducted to explore contributions of biofilm and suspended sludge to nitrogen transformation and nitrous oxide emission in hybrid sequencing batch system |
| (Ma et al., 2015)^17^ | Biofilm reactor | 15 | g N_2_O/kg TN influent | 15 | g N_2_O/kg TN influent | * Laboratory-scale  * To determine the role of inorganic carbon (IC) in influencing the microbial ecology, performance, and nitrogen turnover by individual microbial communities of a biofilm based combined nitritation-anammox process |
| (Kong et al., 2013)^18^ | SBBR (sequencing batch biofilm reactor) | 15 | g N_2_O/kg TN influent | 15 | g N_2_O/kg TN influent | * Laboratory-scale  * The objective of this study was to start-up a laboratory partial nitrification characterizes the emission of N_2_O in an intermittently aerated sequencing batch biofilm reactor |
| (Eldyasti et al., 2014)^19^ | Denitrifying fluidized bed bioreactors | 5.3-15.7 | g N_2_O/kg TN influent | 5.3-15.7 | g N_2_O/kg TN influent | * Laboratory-scale  * To study the influence of biofilm thickness on nitrous oxide (N_2_O) emissions from denitrifying fluidized bed bioreactors |
| (Yang et al., 2013)^20^ | MBBR (moving bed biofilm reactor) | 4.0-20.0 | g N_2_O/kg TN influent | 4.0-20.0 | g N_2_O/kg TN influent | * Two pilot scale reactors |
| (He et al., 2017)^21^ | BAF (biological aerated filter) | 2.4-25.6 | g N_2_O/kg TN influent | 2.4-25.6 | g N_2_O/kg TN influent | * Laboratory-scale  * Three different DO level: 2 mg/L; 4mg/L; 6 mg/L |

Note: the values in brackets indicate the average of emission factors

**Table S3 Summary of CO_2_ emission factors of biological treatment processes from the literature**

| References | Processes | EF in this study | Unit in this study | EF in the literature | Unit in the literature | Unit conversion information or other notes |
| --- | --- | --- | --- | --- | --- | --- |
| (Hu et al., 2019)^22^ | Aerobic biological treatment process | 536-560 (560) | g CO_2_/kg COD removed | Formula only （formula 13） | **-** | * CO_2_, on-site, ana =0.6816*Kd*COD rem; * CO_2_, on-site,cata =0.5088*COD rem; * CO_2_=0.6816*Kd*COD rem+0.5088*COD rem; unit: kg CO_2_/kg COD removed;  * Decay coefficient of domestic wastewater: Kd is0.075 (0.04-0.075) * When Kd=0.04, CO_2_ emission factor=0.536*CODrem; unit: kg CO_2_/kg COD removed * When Kd=0.075, CO_2_ emission factor=0.55992*CODrem; unit: kg CO_2_/kg COD removed |
| (Yan et al., 2014)^3^ | reversed A^2^O | 160.6–294.4 (227.3) | g CO_2_/kg COD removed | 160.6–294.4 (227.3) | g CO_2_/kg COD removed | * Beijing, China; On-site measurement; full scale WWTP emission factor; No sludge related sampling point |
| (Yan et al., 2014)^3^ | A^2^O | 216.2–496.0 (319.3) | g CO_2_/kg COD removed | 216.2–496.0 (319.3) | g CO_2_/kg COD removed | * Beijing, China; On-site measurement; full scale WWTP emission factor; No sludge related sampling point |
| (Bao et al., 2015)^23^ | A^2^O | 580 | g CO_2_/kg COD removed | 580 | g CO_2_/kg COD removed | * Beijing, China; On-site measurement; full scale WWTP emission factor; No sludge related sampling point |
| (Bao et al., 2016)^6^ | SBR | 93.6 | g CO_2_/kg COD removed | 33.5 | g CO_2_/m^3^ wastewater | * The COD and total nitrogen removal efficiencies were higher than 90% and 50% respectively in both the AO and SBR WWTPs  * SBR wastewater inflow: 75.2 * 10^3^m^3^/d  * SBR COD influent was 397.7 mg/L  * SBR TN influent was 62.0 mg/L  * Beijing, China; On-site measurement; full scale WWTP emission factor; No sludge related sampling point |
| (Bao et al., 2015)^23^ | SBR | 970 | g CO_2_/kg COD removed | 970 | g CO_2_/kg COD removed | * Beijing, China; On-site measurement; full scale WWTP emission factor; No sludge related sampling point |
| (Yan et al., 2014)^3^ | OD | 146.2–377.5 (261.3) | g CO_2_/kg COD removed | 146.2–377.5 (261.3) | g CO_2_/kg COD removed | * Beijing, China; On-site measurement; full scale WWTP emission factor; Sludge related sampling point: sludge thickener and sludge storage area |
| (Bao et al., 2015)^23^ | OD | 760 | g CO_2_/kg COD removed | 760 | g CO_2_/kg COD removed | * Beijing, China; On-site measurement; full scale WWTP emission factor; Sludge related sampling point: sludge distribution tank and sludge thickening tank |
| (Bao et al., 2015)^23^ | AO | 680 | g CO_2_/kg COD removed | 680 | g CO_2_/kg COD removed | * Beijing, China; On-site measurement; full scale WWTP emission factor; No sludge related sampling point |
| (Bao et al., 2016)^6^ | AO | 51.5 | g CO_2_/kg COD removed | 16.91 | g CO_2_/m^3^ wastewater | * The COD and total nitrogen removal efficiencies were higher than 90% and 50% respectively in both the AO and SBR WWTPs  * AO COD influent was 364.8 mg/L  * Emission factor was 16.91/ (0.9*364.8) =51.5 g CO_2_/kg COD removed  * Beijing, China; On-site measurement; full scale WWTP emission factor; No sludge related sampling point |
| (Hu et al., 2019)^22^ | Biofilm process | 436.2-958.6 (436.2) | g CO_2_/kg COD removed | Formula only | - | * on-site CO_2_ emission of biofilm treatment process= CO_2_, on-site, ana+ CO_2_, on-site,cata; * CO_2_, on-site, ana =1.42*b'*Y*0.8* COD rem; unit: kg CO_2_/kg COD removed * CO_2_, on-site,cata =1.59* (1-Y)*0.8*COD rem; unit: kg CO_2_/kg COD removed * Y：0.8 (0.3-0.8); Y: sludge yield ratio * b'：0.2; b': overall biofilm specific loss rate * when Y=0.3, emission factor is 0.9586*CODrem kg CO_2_/kg COD removed * when Y=0.8, emission factor is 0.4362*CODrem kg CO_2_/kg COD removed |
| (Hu et al., 2019)^22^ | Anaerobic biological treatment | 266.3-494.6 (380.5) | g CO_2_/kg COD removed | Formula only | - | * Mathematical model (formula 19) * In formula 19, Y_CO2_=29% is the theoretical yield ratio of CO_2_, we assume the uncertainty of Y_CO2_ is ±30%, therefore Y_CO2_ is 20.3%-37.7%  * CO_2_=1.312*Y_CO2_*CODrem; Y_CO2_=20.3%-37.7% (29%) |

Note: the values in brackets indicate the average of emission factors

**Table S4 The uncertainty of total GHG emissions of WWTPs**

|  | Low | Up |
| --- | --- | --- |
| 2006 | -27% | 81% |
| 2007 | -27% | 81% |
| 2008 | -26% | 80% |
| 2009 | -27% | 83% |
| 2010 | -23% | 87% |
| 2011 | -24% | 93% |
| 2012 | -24% | 91% |
| 2013 | -24% | 93% |
| 2014 | -23% | 92% |
| 2015 | -23% | 95% |
| 2016 | -23% | 97% |
| 2017 | -23% | 97% |
| 2018 | -23% | 97% |
| 2019 | -23% | 92% |

**Table S5 Comparing wastewater GHG estimations in this study with existing research**

| References | Type of wastewater | GHG | Prefectural/Provincial/National level | Year | Emission factors | Activity Data | Accounting results in references | Accounting results in this study |
| --- | --- | --- | --- | --- | --- | --- | --- | --- |
| (Zhao et al., 2019)^24^ | Wastewater from Municipal Wastewater Treatment Plants (MWWTPs) | CH_4_ | Prefectural level (studied 229 prefectural level cities out of 288 in mainland China) | 2014 | * IPCC 2006 approach * MCF: 0 for well-managed aerobic systems; 0.3 for not well-managed aerobic systems, and 0.8 for anaerobic systems * for those WWTPs with no data on their treatment processes, MCF is 0.165 | Activity data of 2019 WWTPs are from Urban Drainage Statistic Yearbook | CH_4_ emissions in 2014: 29.2 Mt CO_2_eq | CH_4_ emissions from MWWTPs and other facilities in 2014: 2.55 (1.79-4.13) Mt CO_2_eq; |
| (Yan et al., 2019)^25^ | Municipal Wastewater Treatment Plants，only domestic wastewater | CH_4_, N_2_O | Provincial level (34 provincial level regions in China) | 2005-2014 | * Emission factors are from studies of actual MWWTPs in China * CH_4_ emission factor: 2.3064 kg CH_4_/t COD removed； * N_2_O emission factor: 2.0794 kg N_2_O /t TN removed | * China Environmental Statistic Yearbooks * China Statistical Yearbook * The Food and Agriculture Organization (FAO) | * CH_4_ emissions in 2014: 769.08 Gg CO_2_ eq in 2014 (0.769 Mt CO_2_eq) * N_2_O emissions in 2014: 524.95 Gg CO_2_ eq in 2014 (0.524 Mt CO_2_eq) | * CH_4_ emissions from MWWTPs and other facilities in 2014: 2.55 (1.79-4.13) Mt CO_2_eq; * N_2_O emissions from MWWTPs and other facilities in 2014: 3.9 (2.20-9.91) Mt CO_2_eq; |
| (Du et al., 2018)^26^ | Domestic and industrial wastewater | CH_4_ | Provincial level (30 province-level regions in China) | domestic wastewater (2000-2009) industrial wastewater (2000-2014) | * IPCC 2006 approach and NDRC 2007 approach * MCF is 0.165 for domestic wastewater * MCF is 0.458 for industrial wastewater * MCF is 0.1 for wastewater discharged into rivers, lakes, and oceans | * China Environmental Statistic Yearbooks | * CH_4_ emissions from industrial wastewater in 2014: about 2600 Gg/year (industrial: 72.8 Mt CO_2_eq/y) * CH_4_ emissions from domestic wastewater in 2014: about 800 Gg/year (domestic: 22.4 Mt CO_2_eq/y) | CH_4_ emissions from MWWTPs and other facilities in 2014: 2.55 (1.79-4.13) Mt CO_2_eq; |
| (Peng et al., 2016)^27^ | Domestic and industrial wastewater | CH_4_ | National level | 1980-2010 | * IPCC 2006 approach * MCF is 0.165 for domestic wastewater; * MCF is 0.467 for industrial wastewater; * MCF is 0.1 for wastewater discharged into rivers, lakes, and oceans | * China Statistical Yearbooks | * CH_4_ emissions from industrial and domestic wastewater in total in 2010 are 2.3 (1.2-2.6) Tg CH_4_/year (64.4(33.6-72.8) Mt CO_2_eq/y) | CH_4_ emissions from MWWTPs and other facilities in 2010: 1.71 (1.22-2.73) Mt CO_2_eq |
| (Zhang and Chen, 2014) ^28^ | Domestic and industrial wastewater | CH_4_ | Provincial level (31 provincial level regions in China) | 2007 | * MCF is 0.165 for domestic wastewater * MCF is 0.4674 for industrial wastewater * MCF is 0.1 for wastewater discharged into water bodies | * China Environmental Statistic Yearbooks | * CH_4_ emissions from domestic wastewater in 2007 :564 Gg (15.8 Mt CO_2_eq) | CH_4_ emissions from MWWTPs and other facilities in 2007: 0.58 (0.27-1.22) Mt CO_2_eq |
| (Ma et al., 2015)^29^ | Domestic and industrial wastewater | CH_4_ | National level | 2005-2010 | * IPCC 1996 approach and IPCC Good Practice Guidance and Uncertainty Management in National Greenhouse Gas Inventories * MCF is 0.165 for domestic wastewater * MCF is 0.458 for industrial wastewater | * China Statistics Yearbook * China Environment Statistics Yearbook | * CH_4_ emissions from the treatment of domestic wastewater were 0.6110 Mt (17.11 Mt CO_2_eq) in 2010 | CH_4_ emissions from MWWTPs and other facilities in 2010: 1.71 (1.22-2.73) Mt CO_2_eq |
| (NDRC, 2018)^30^ | Domestic and industrial wastewater | CH_4_, N_2_O | National level | 2010 | IPCC 1996 and IPCC 2006 approach | * China Environment Statistics Yearbook 2010 * China Environmental Statistics Annual Report 2010 * Municipal wastewater treatment plants compilation in China | * CH_4_ emissions from industrial and domestic wastewater in total in 2010: 2.194 Mt CH_4_/year (61.4 Mt CO_2_eq/year) in 2010 * N_2_O emissions from industrial and domestic wastewater in total in 2010: 0.096 Mt N_2_O /year (25.4 Mt CO_2_eq/year) in 2010 | * CH_4_ emissions from MWWTPs and other facilities in 2010: 1.71 (1.22-2.73) Mt CO_2_eq; * N_2_O emissions from MWWTPs and other facilities in 2010: 2.92 (1.85-6.45) Mt CO_2_eq |
| (NDRC, 2018)^31^ | Domestic and industrial wastewater | CH_4_, N_2_O | National level | 2014 | * IPCC 2006 approach  * IPCC 2006 default emission factors | China Environment Statistics Yearbook 2014 | * CH_4_ emissions from industrial and domestic wastewater in total in 2014: 2.721 Mt CH_4_/year (76.2 Mt CO_2_eq/year) in 2014; * N_2_O emissions from industrial and domestic wastewater in total in 2014: 0.11 Mt N_2_O /year (29.2 Mt CO_2_eq/year) in 2014 | * CH_4_ emissions from MWWTPs and other facilities in 2014: 2.55 (1.79-4.13) Mt CO_2_eq; * N_2_O emissions from MWWTPs and other facilities in 2014: 3.9 (2.20-9.91) Mt CO_2_eq |
| (Guo et al 2019)^32^ | Wastewater from Municipal Wastewater Treatment Plant | CH_4_, N_2_O from biological treatment and CO_2_ from electricity consumption | Prefectural level | 2007-2016 | * IPCC 2019 approach | *Statistical Yearbook of Urban Drainage  * Firm level data | * Total emissions of CH_4_ and N_2_O from biological treatment and CO_2_ from electricity in 2014, 2015 and 2016 were: 28.0, 29.8 and 31.4 Mt CO_2_eq | Total emissions of CH_4_ and N_2_O from biological treatment and CO_2_ from electricity in 2014, 2015 and 2016 were: 15.9, 16.0 and 15.8 Mt CO_2_eq |

# CO_2_ emission factors of effluent discharge

According to the ‘Annex 6A.4 Calculation of MCF for methane emissions from sewage discharges’ on page 6.59 and page 6.60 of IPCC 2019, we derived CO_2_ emission factors from wastewater discharges. Firstly, we obtained CO_2_ yield for rivers, lakes, and reservoirs from TABLE 6A.3 (NEW) on page 6.60 of IPCC 2019 by subtracting CH_4_ yield from 100%. Table S7 lists CO_2_ yield for rivers, lakes, and reservoirs.

In the Annex 6A.4 of IPCC 2019, we know that the COD of glucose on a carbon basis is 2.67 kg COD/C. Therefore, taking the minimum CO_2_ yield 73% (0.73 CO_2_-C/kg (CH_4_-C+ CO_2_-C))as an example, 0.73 CO_2_-C/kg (CH_4_-C+ CO_2_-C) corresponds to 0.273 kg CO_2_-C/kg COD, which was calculated by (0.73 [CO_2_-C/kg (CH_4_-C+CO_2_-C)]÷2.67 [kg COD/C]=0.273 kg CO_2_-C/kg COD. In IPCC 2019, 50% carbon inputs were assumed to be emitted to the atmosphere, and the remainder were sequestered in sediments or lost to the ocean. Thus, CO_2_ emission factor is 0.273 [kg CO_2_-C/kg COD] *(12+16*2)/12 [kg CO_2_/kg CO_2_-C] *50%= 0.501 kg CO_2_/kg COD.

From Annex 6A.4, the equation to calculate CH_4_ emission factor from sewage discharges is as follows:

$${EF}_{CH4} \left( \frac{kg {CH}_{4}}{kg COD} \right)={yield}_{CH4} \left( \frac{kg {CH}_{4}-C}{kg {CH}_{4}-C+kg CO2-C} \right)\div2.67 \left( \frac{kg COD}{kg C} \right)*\frac{12+4}{12}\left( \frac{kg {CH}_{4}}{kg {CH}_{4}-C} \right)*0.5$$

While CO_2_ emission factor is calculated as:

$${EF}_{CO2}\left( \frac{kg {CO}_{2}}{kg COD} \right)={yield}_{CO2} \left( \frac{kg {CO}_{2}-C}{kg {CH}_{4}-C+kg {CO}_{2}-C} \right)\div2.67 \left( \frac{kg COD}{kg C} \right)*\frac{12+16*2}{12}\left( \frac{kg {CO}_{2}}{kg {CO}_{2}-C} \right)*0.5$$

**Table S7 Yield and emission factor of CO_2_ for rivers, lakes, and reservoirs**

| Reference | Type of water body | n (reservoirs, lakes or rivers) | CH_4_ Approx. revised yield | CO_2_ Approx. revised yield | CH_4_ emission factor (kg CH_4_/kg COD) | CO_2_ emission factor (kg CO_2_/kg COD) |
| --- | --- | --- | --- | --- | --- | --- |
| (Deemer et al., 2016)^33^ | Reservoirs | 75 | 27% | 73% | 0.0675 | 0.501 |
| (Bastviken et al., 2011)^34^;  (St. Louis et al., 2000)^35^ | Reservoirs | 161 | 20%-22% | 78%-80% | 0.05-0.055 | 0.536-0.549 |
| (Barros et al., 2011)^36^; (Li and Zhang, 2014)^37^ | Hydroelectric Reservoirs | 85; 104 | 8%-20% | 80%-92% | 0.02-0.05 | 0.549-0.632 |
| (Bastviken et al., 2011)^34^;  (Raymond et al., 2013)^38^ | Lakes | 66 | 19% | 81% | 0.0475 | 0.556 |
| (Bastviken et al., 2011)^34^;  (Stanley et al., 2016)^39^; (Lauerwald et al., 2015)^40^ | Rivers | 21; 26 | 0.39%-6% | 94%-99.61% | 0.001-0.015 | 0.645-0.684 |

Therefore, the minimum and maximum CO_2_ emission factor for reservoirs, lakes or rivers are 0.501 and 0.684 kg CO_2_/kg COD. The average value is calculated by:

[0.501+(0.536+0.549)/2+(0.549+0.632)/2+0.556+(0.645+0.684)/2]/5=0.5709 (kg CO_2_/kg COD)

# References

1. Aboobakar, A., Jones, M., Vale, P., Cartmell, E. & Dotro, G. Methane Emissions from Aerated Zones in a Full-Scale Nitrifying Activated Sludge Treatment Plant. *Water Air Soil Pollut* **225**, 1814 (2013).

2. Ren, Y. g. *et al.* Nitrous oxide and methane emissions from different treatment processes in full-scale municipal wastewater treatment plants. *null* **34**, 2917–2927 (2013).

3. Yan, X., Li, L. & Liu, J. Characteristics of greenhouse gas emission in three full-scale wastewater treatment processes. *Journal of Environmental Sciences* **26**, 256–263 (2014).

4. Liu, Y., Cheng, X., Lun, X. & Sun, D. CH4 emission and conversion from A^2^O and SBR processes in full-scale wastewater treatment plants. *Journal of Environmental Sciences* **26**, 224–230 (2014).

5. Wang, J. *et al.* Methane emissions from a full-scale A/A/O wastewater treatment plant. *Bioresource Technology* **102**, 5479–5485 (2011).

6. Bao, Z., Sun, S. & Sun, D. Assessment of greenhouse gas emission from A/O and SBR wastewater treatment plants in Beijing, China. *International Biodeterioration & Biodegradation* **108**, 108–114 (2016).

7. Czepiel, Peter., Crill, Patrick. & Harriss, Robert. Nitrous Oxide Emissions from Municipal Wastewater Treatment. *Environ. Sci. Technol.* **29**, 2352–2356 (1995).

8. Brotto, A. C. *et al.* Factors controlling nitrous oxide emissions from a full-scale activated sludge system in the tropics. *Environ Sci Pollut Res* **22**, 11840–11849 (2015).

9. Sun, S. *et al.* N2O emission from full-scale urban wastewater treatment plants: a comparison between A^2^O and SBR. *Water Science and Technology* **67**, 1887–1893 (2013).

10. Wang, J. *et al.* Nitrous oxide emissions from a typical northern Chinese municipal wastewater treatment plant. *Desalination and Water Treatment* **32**, 145–152 (2011).

11. Wang, Y. *et al.* Nitric oxide and nitrous oxide emissions from a full-scale activated sludge anaerobic/anoxic/oxic process. *Chemical Engineering Journal* **289**, 330–340 (2016).

12. Sun, S., Cheng, X. & Sun, D. Emission of N2O from a full-scale sequencing batch reactor wastewater treatment plant: Characteristics and influencing factors. *International Biodeterioration & Biodegradation* **85**, 545–549 (2013).

13. Sun, S., Bao, Z. & Sun, D. Study on emission characteristics and reduction strategy of nitrous oxide during wastewater treatment by different processes. *Environ Sci Pollut Res* **22**, 4222–4229 (2015).

14. Sun, S. *et al.* Reduction and prediction of N2O emission from an Anoxic/Oxic wastewater treatment plant upon DO control and model simulation. *Bioresource Technology* **244**, 800–809 (2017).

15. Wang, Y., Fang, H., Zhou, D., Han, H. & Chen, J. Characterization of nitrous oxide and nitric oxide emissions from a full-scale biological aerated filter for secondary nitrification. *Chemical Engineering Journal* **299**, 304–313 (2016).

16. Lo, I. W., Lo, K. V., Mavinic, D. S., Shiskowski, D. & Ramey, W. Contributions of biofilm and suspended sludge to nitrogen transformation and nitrous oxide emission in hybrid sequencing batch system. *Journal of Environmental Sciences* **22**, 953–960 (2010).

17. Ma, Y., Sundar, S., Park, H. & Chandran, K. The effect of inorganic carbon on microbial interactions in a biofilm nitritation–anammox process. *Water Research* **70**, 246–254 (2015).

18. Kong, Q. *et al.* Partial nitrification and nitrous oxide emission in an intermittently aerated sequencing batch biofilm reactor. *Chemical Engineering Journal* **217**, 435–441 (2013).

19. Eldyasti, A., Nakhla, G. & Zhu, J. Influence of biofilm thickness on nitrous oxide (N2O) emissions from denitrifying fluidized bed bioreactors (DFBBRs). *Journal of Biotechnology* **192**, 281–290 (2014).

20. Yang, J., Trela, J., Plaza, E. & Tjus, K. N2O emissions from a one stage partial nitrification/anammox process in moving bed biofilm reactors. *Water Science and Technology* **68**, 144–152 (2013).

21. He, Q. *et al.* Impact of dissolved oxygen on the production of nitrous oxide in biological aerated filters. *Front. Environ. Sci. Eng.* **11**, 16 (2017).

22. Hu, W., Tian, J. & Chen, L. Greenhouse gas emission by centralized wastewater treatment plants in Chinese industrial parks: Inventory and mitigation measures. *Journal of Cleaner Production* **225**, 883–897 (2019).

23. Bao, Z., Sun, S. & Sun, D. Characteristics of direct CO2 emissions in four full-scale wastewater treatment plants. *Desalination and Water Treatment* **54**, 1070–1079 (2015).

24. Zhao, X. *et al.* China’s urban methane emissions from municipal wastewater treatment plant. *Earth’s Future* **7**, 480–490 (2019).

25. Yan, X. *et al.* Spatial and Temporal Distribution of Greenhouse Gas Emissions From Municipal Wastewater Treatment Plants in China From 2005 to 2014. *Earth’s Future* **7**, 340–350 (2019).

26. Du, M. *et al.* Estimates and Predictions of Methane Emissions from Wastewater in China from 2000 to 2020. *Earth’s Future* **6**, 252–263 (2018).

27. Peng, S. *et al.* Inventory of anthropogenic methane emissions in mainland China from 1980 to 2010. *Atmos. Chem. Phys.* **16**, 14545–14562 (2016).

28. Zhang, B. & Chen, G. Q. Methane emissions in China 2007. *Renewable and Sustainable Energy Reviews* **30**, 886–902 (2014).

29. Ma, Z.-Y. *et al.* CH4 emissions and reduction potential in wastewater treatment in China. *Advances in Climate Change Research* **6**, 216–224 (2015).

30. NDRC. *The People’s Republic of China Third National Communication on Climate Change*. http://tnc.ccchina.org.cn/archiver/NCCCcn/UpFile/Files/Htmleditor/202007/20200723152332694.pdf (2018).

31. NDRC. *The People’s Republic of China Second Biennial Update Report on Climate Change*. http://tnc.ccchina.org.cn/archiver/NCCCcn/UpFile/Files/Htmleditor/202007/20200723155226725.pdf (2018).

32. Guo, S., Huang, H., Dong, X. & Zeng, S. Calculation of greenhouse gas emissions of municipal wastewater treatment and its temporal and spatial trend in China. *Water & wastewater engineering* **45**, 56–62 (2019).

33. Deemer, B. R. *et al.* Greenhouse Gas Emissions from Reservoir Water Surfaces: A New Global Synthesis. *BioScience* **66**, 949–964 (2016).

34. Bastviken, D., Tranvik, L. J., Downing, J. A., Crill, P. M. & Enrich-Prast, A. Freshwater Methane Emissions Offset the Continental Carbon Sink. *Science* **331**, 50–50 (2011).

35. St. Louis, V. L., Kelly, C. A., Duchemin, É., Rudd, J. W. M. & Rosenberg, D. M. Reservoir Surfaces as Sources of Greenhouse Gases to the Atmosphere: A Global Estimate: Reservoirs are sources of greenhouse gases to the atmosphere, and their surface areas have increased to the point where they should be included in global inventories of anthropogenic emissions of greenhouse gases. *BioScience* **50**, 766–775 (2000).

36. Barros, N. *et al.* Carbon emission from hydroelectric reservoirs linked to reservoir age and latitude. *Nature Geosci* **4**, 593–596 (2011).

37. Li, S. & Zhang, Q. Carbon emission from global hydroelectric reservoirs revisited. *Environ Sci Pollut Res* **21**, 13636–13641 (2014).

38. Raymond, P. A. *et al.* Global carbon dioxide emissions from inland waters. *Nature* **503**, 355–359 (2013).

39. Stanley, E. H. *et al.* The ecology of methane in streams and rivers: patterns, controls, and global significance. *Ecological Monographs* **86**, 146–171 (2016).

40. Lauerwald, R., Laruelle, G. G., Hartmann, J., Ciais, P. & Regnier, P. A. G. Spatial patterns in CO2 evasion from the global river network. *Global Biogeochemical Cycles* **29**, 534–554 (2015).
